# Identification of Microorganisms by High Resolution Tandem Mass Spectrometry with Accurate Statistical Significance

**DOI:** 10.1007/s13361-015-1271-2

**Published:** 2015-10-28

**Authors:** Gelio Alves, Guanghui Wang, Aleksey Y. Ogurtsov, Steven K. Drake, Marjan Gucek, Anthony F. Suffredini, David B. Sacks, Yi-Kuo Yu

**Affiliations:** National Center for Biotechnology Information, National Library of Medicine, National Institutes of Health, Bethesda, MD 20894 USA; Proteomics Core, National Heart, Lung, and Blood Institute, National Institutes of Health, Bethesda, MD 20892 USA; Critical Care Medicine Department, Clinical Center, National Institutes of Health, Bethesda, MD 20892 USA; Department of Laboratory Medicine, Clinical Center, National Institutes of Health, Bethesda, MD 20892 USA

**Keywords:** Pathogen identification, Microorganism classification, Statistical significance, Mass spectrometry, Proteomics

## Abstract

Correct and rapid identification of microorganisms is the key to the success of many important applications in health and safety, including, but not limited to, infection treatment, food safety, and biodefense. With the advance of mass spectrometry (MS) technology, the speed of identification can be greatly improved. However, the increasing number of microbes sequenced is challenging correct microbial identification because of the large number of choices present. To properly disentangle candidate microbes, one needs to go beyond apparent morphology or simple ‘fingerprinting’; to correctly prioritize the candidate microbes, one needs to have accurate statistical significance in microbial identification. We meet these challenges by using peptidome profiles of microbes to better separate them and by designing an analysis method that yields accurate statistical significance. Here, we present an analysis pipeline that uses tandem MS (MS/MS) spectra for microbial identification or classification. We have demonstrated, using MS/MS data of 81 samples, each composed of a single known microorganism, that the proposed pipeline can correctly identify microorganisms at least at the genus and species levels. We have also shown that the proposed pipeline computes accurate statistical significances, i.e., *E*-values for identified peptides and unified *E*-values for identified microorganisms. The proposed analysis pipeline has been implemented in MiCId, a freely available software for Microorganism Classification and Identification. MiCId is available for download at http://www.ncbi.nlm.nih.gov/CBBresearch/Yu/downloads.html.

**Electronic supplementary material:**

The online version of this article (doi:10.1007/s13361-015-1271-2) contains supplementary material, which is available to authorized users.

**Graphical Abstract:**
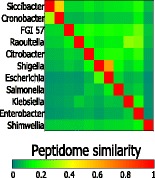
ᅟ

## Introduction

Correct and rapid identification of microorganisms is the key to the success of many important applications in health and safety, including, but not limited to, infection treatment, food safety, and biodefense. State-of-the-art technologies for microbial identification include both next generation sequencing and mass spectrometry (MS). The former method, although being a newer technology and having great success, generally needs extensive sample preparations and a considerable amount of data analysis time [1, 2]. On the other hand, several studies have demonstrated the capability of MS-based technology in identifying microorganisms with a high degree of accuracy [3–6]. This technology is being employed to rapidly identify pathogens in clinical settings, to improve food safety by detecting bacterial contaminants, to detect pathogens relevant to biodefense, to assist in the identification of novel microorganisms, and to classify microorganisms phylogenetically [7–12]. The idea of using MS-based technology to identify microorganisms dates back to 1970s [13–15]. However, it was not until sufficient progress was made—in the fields of MS, DNA sequencing and bioinformatics—that MS-based methods became practical for identifying microorganisms [16–22].

There are different methods that employ MS-based technology to identify pathogens. Of these methods, matrix-assisted laser desorption/ionization (MALDI)-based systems [23–25] and polymerase chain reaction electrospray ionization mass spectrometry (PCR-ESI-MS)-based systems have been the focus of most research in this direction [26–29]. Comparison between these two systems in terms of their ability to accurately identify microorganisms has been performed with no significant difference found, both having about 95% identification accuracy at the species level [4]. Evaluations of microbial identification accuracy of different MALDI-based systems have also reported comparable performance [5, 6]. In the next two paragraphs, we briefly summarize the PCR-ESI-MS- and MALDI-based systems; the listed citations therein provide more detailed and comprehensive descriptions of both systems.

The PLEX-ID system produced by Abbot Molecular was the main commercially available PCR-ESI-MS-based system; it is also known as the T5000 system in earlier publications [26–28]. Abbot discontinued the manufacturing of the PLEX-ID system in 2012, although it continues to be used in many research labs [30, 31]. Sample preparation for PCR-ESI-MS-based systems requires extracting nucleic acids from clinical specimens or from cultivated microbial isolates [29, 30, 32]. The extracted DNA is then transferred into a 96-well plate, where each well usually contains a single set of broad-range PCR primers for DNA amplification. After PCR amplification, the sample is desalted and sent to a mass spectrometer where the mass over charge (*m/z*) of the amplicons are measured [9]. Microbial identification is done using a commercial software that makes microbial inferences based on the following assumptions: genetic targets must be present for primers used, a small number of possible base compositions must be associated with a measured amplicon *m/z*, and observed amplicons *m/z* values must have matches for the designed genetic primer regions in a microbial DNA database. If all the above assumptions are satisfied, this is a robust technology. Some, however, criticize the small number of amplicons measured and caution the possibility of the formation of chimeric DNA, especially when analyzing a sample made of more than one microorganism [11, 29, 33].

Two main commercially available MALDI-based systems are the BioTyper (Bruker Daltonics, Bremen, Germany) and the VITEX MS (BioMérieux, Marcy l′Etoile, France). Advantages of these systems include simple operation, low cost, and short time for sample analysis. For microbial identification, one needs a purified microbial culture, which is then mixed with an absorbing organic acid (matrix); the mixture is then allowed to air dry and it is finally placed in the MALDI-TOF mass spectrometer [3, 23–25, 34]. Ionizing the mixture by laser, the MALDI-TOF system produces the corresponding *m/z* spectrum and queries it against a MALDI-TOF mass spectral database [35] for identification (i.e., using the spectrum as a fingerprint for the underlying microorganism). Even though MALDI-based systems yield reproducible and accurate microbial identifications [3, 23–25, 34, 36], there remain areas that can be improved upon. For example, there is a need for statistical significance assignment in fingerprint matching [37]. Also, even though sample preparation methods have been standardized [38], the optimal protocol appears to vary by microorganism [37]. Further, growth medium seems to affect identification specificity [39]; significant mass fingerprint fluctuations have been observed for filamentous fungi because of changes in culture conditions [40]. Another challenging issue for MALDI-based systems pertains to polymicrobial culture attributable to complex infections or contaminants [9].

In this manuscript, we present an analysis pipeline for microbial identification or taxonomic classification using tandem MS (MS/MS) spectra as input. To facilitate reading, we have provided an acronym list in Table 1. All proteomics data used are produced by high resolution mass analyzers [41, 42], yielding high mass accuracy for both precursor and product ions. Mass accuracy of these instruments in daily operation can range from 1 to 10 parts per million (ppm) depending on several factors [42]. Higher mass accuracy is desirable because more accurate assignment of charge and mass to precursor and product ions can be achieved, thus leading to a better sensitivity in peptide identification. Liquid chromatography MS (LCMS) experiments [43] can be viewed as complementary or as orthogonal to the MALDI- and PCR-ESI-MS-based systems. While MALDI-based systems provide a fingerprint of a microorganism’s ionized cells and PCR-ESI-MS-based systems supply the *m/z* of a limited number of selected regions of a microorganism’s genome, the data obtained by LCMS experiments produce hundreds to thousands of confidently identified peptides (CIPs) of a microorganism’s peptidome. Containing a rich array of information, the CIPs, after proper analyses, can be used to identify/classify microbes directly or in conjunction with other approaches.

**Table 1:** List of Commonly Used Acronyms

**Acronym**	**Definition**
CIP	confidently identified peptide
CI	cluster index
*E*	*E*-value
*E*_*u*_	unified *E*-value
E[X]	expected value of variable X
*E*_*c*_	the *E*-value that E[FP] ≤ 1
FP	false positives
IF	identification fraction
NIP	number of identified peptides
NUP	number of unique peptides
MWET	molecular weight error tolerance
*n*_*s*_	number of MS/MS spectra from a given sample
*n*_*mw*_	number of qualified peptides in the database
MCS	missed cleavage sites
OD	optical density
P	*P*-value
*P*_*u*_	unified *P*-value
ppm	parts per million
PNNL	Pacific Northwest National Laboratory
R	rank
SN	sample number
SSE	statistically significant *E*-value
WPC	weighted peptide count

Although several studies have demonstrated the usefulness of MS/MS data in the identification of microorganisms [44–47], only a few proposed computational methods are specifically designed to perform microbial identification using MS/MS data. An existing method infers microbial identification [48, 49] based on confident identifications of peptides specific to certain microorganisms. This approach, however, might not be pragmatic because one needs to construct a set of unique and experimentally detectable peptides for each microorganism, and this set must be continuously checked for uniqueness as protein sequences from new microorganisms become available. Also, the presence of these unique peptides might be questioned when microorganisms are cultured in different media [39]. There also exists another approach [50] that utilizes a set of CIPs from a LCMS experiment. This approach uses a mixture model that was learned from a training dataset to compute posterior probabilities. For each MS/MS spectrum, the posterior probability of the best ranked peptide being a true positive is computed. In this approach, a microorganism having the highest number of matched CIPs is considered to be the correct identification [50]. A later development extended the scope to include microbial classification by generating a binary matrix, where a value of 1 is assigned if a peptide belongs to a microorganism and 0 otherwise [51].

The analysis pipeline developed in this manuscript exhibits similarities to all the aforementioned methods [48–51], but it also differs from them in several fundamental aspects. First, all identified peptides are considered in our approach. This is important because a peptide’s fragmentation series currently used by database search tools’ scoring functions are learned empirically and collectively for all peptides rather than theoretically computed for each peptide [52]. Therefore, there can be cases when the score differences among the top ranking peptides are small, and utilizing only the best ranked identified peptide per MS/MS spectrum might not be the best approach to take. Second, our approach is built on a MS/MS spectrum-specific measure, namely, *E*-value, which is computed per MS/MS spectrum for all identified peptides [53, 54]. Evidently, using a measure such as *E*-value or *P*-value that takes into account spectrum specificity is more robust against cross-spectrum or cross-experiment variations than utilizing an unnormalized measure [55]. For example, if an unnormalized measure, such as score, is used, a peptide identified with score 3 from MS/MS spectrum A can, in principle, signify a better identification than another peptide identified with score 3.5 from MS/MS spectrum B. By using the *E*-value, one can avoid such a problem: one can simply compare and combine identified peptides across MS/MS spectra and even across different experiments. Third, statistical significance in the form of a unified *E*-value (*E*_*u*_) is computed and assigned to identified microorganisms. An *E*_*u*_ is computed by combining the *E*-values of a microorganism’s CIPs [56] (i.e., whose *E*-values fall below the cutoff set by demanding the expected number of false positive (FP) peptides included in the analysis be <1).

In summary, in this manuscript we present an analysis pipeline that uses MS/MS spectra for microbial identification and/or classification. Interpretation of the results depends on the presence/absence of the correct microorganism in the database. If we are certain that the correct microorganism is present in the database, we should interpret the results as microbial identification. On the other hand, if we are sure that the correct microorganism is absent from the database, we may interpret the results as microbial classification. We have demonstrated, using MS/MS data of 81 samples, each composed of a single known microorganism, that the proposed pipeline can correctly identify microorganisms at least at the genus and species levels. We have also shown that the proposed pipeline computes accurate statistical significances (i.e., *E*-values for identified peptides and *E*_*u*_ for identified microorganisms). The proposed analysis pipeline has been implemented in MiCId, a freely available software for Microorganism Classification and Identification.

## Materials and Methods

### In-House Dataset

#### Bacterial culture preparation: batch one

Fresh *Escherichia coli* (ATCC 25922) and *Pseudomonas aeruginosa* (ATCC 27853) plates were used to inoculate a 2 mL tryptic broth for overnight growth. From each saturated culture, seven 2 mL vials were inoculated with 20 μL (1:100 dilution) and put in shaker at 37°C. The rest of the overnight culture was used for the saturated time point. Each culture growth was monitored by nephelometer and recorded in Table 2. To have approximately the same number of cells in each sample, four tubes were combined for the low time point and two tubes for the medium time point. One tube was used for the high time point. Serial additions of each time point were added to two Eppendorf tubes and spun at 14 K rpm for 2 min until all of the sample was in the Eppendorf tube and the supernatants discarded. These pellets were washed with 1 mL 70% EtOH and then resuspended in 150 μL 70% formic acid. After vortexing, 150 μL acetonitrile was added and samples were vortexed and respun. Supernatants of each pair of tubes were combined to create eight samples (*E. coli*, *P. aeruginosa*) × Low, Medium, High, Saturated) with 600 μL each. Each sample was divided into four tubes and speed-vacuumed to dry. Two sets of these tubes were then digested. To each tube, 40 μL of 5 M Gnd HCL and 25 mM NH_4_HCO_3_ was added, and the tube was sonicated for 45 min with occasional vortexing. Samples were reduced with DTT (2 μL1 M in water, 37°C for 60 min), alkylated (10 μL iodoacetamide 40 mg/mL in water, at room temperature for 60 min in the dark), and quenched with D TT (2 μL, 15 min). The tubes were neutralized by the addition of 200 μL 25 mM NH_4_HCO_3_ containing 1 μL trypsin (Promega). Samples were digested using the CEM Discovery microwave digester (15 min, 56°C). After digestion, samples were stored at –20°C until used.

**Table 2:** Monitor Culture Growth

**Batch one Samples**					
Time (h)	Sample label	Number vials	OD^a^ *E. coli*	OD *P. aeruginosa*	
0:00			0.03	0.03	
2:00	Low	4	0.30	0.38	
3:30	Medium	2	0.75	0.65	
5:00	High	1	1.07		
6:00	High	1		0.90	
14:00	Saturated	1	1.34	1.50	
**Batch two Samples**					
Time (h)	Sample label	Number vials	OD^a^ *E. coli*	OD *P. aeruginosa*	OD *S. enterica*
0:00			0.03	0.03	0.03
2:10	Low	4	0.34	0.43	0.42
3:00	Medium	2	0.66		0.68
3:40	Medium	2		0.64	
5:40	High	1	1.01		
7:10	High	1		1.12	0.96
14:00	Saturated	1	1.34	1.50	1.34
**Batch three Samples**					
time (h)	Sample label	Number vials	OD^a^ *E. coli*	OD *P. aeruginosa*	
3:00–4:00	Medium	4	0.6–0.7	0.6–0.7	

^a^ Optical density (OD) 0.39 ≈ 8 × 10^8^ cells. Roughly linear for OD between 0 and 0.40

#### Bacterial culture preparation: batches two and three

In addition to samples of *Escherichia coli* (ATCC 25922) and *Pseudomonas aeruginosa* (ATCC 27853), the second batch also contains samples from *Salmonella enterica subspecies serovar Typhimurium* (SL1344). In terms of sample preparation, all three batches largely follow the same procedures except for steps indicated by the underlined text in the previous subsection. For batches two and three, the aforementioned underlined steps should be replaced by “Tubes were diluted by the addition of 200 μL 50 mM NH_4_HCO_3_, then ProteaseMAX surfactant (3 μL of a 1% solution in 100 μL 50 mM NH_4_HCO_3_, Promega) was added to 0.01% final concentration and mixed gently. Trypsin was then added (2 μL of 500 μg/mL in 50 mM HOAc).” In addition to the number of microorganisms used, there is another small difference between batch two and batch three: samples from batch three were all of the medium growth range.

### Liquid Chromatography-Tandem Mass Spectrometry (LC-MS/MS) Acquisition

LC/MS-MS was performed using an Eksigent nanoLC-Ultra 2D system (Dublin, CA, USA) coupled to an Orbitrap Elite mass spectrometer (Thermo Scientific, San Jose, CA, USA). Twenty percent of each peptide sample was first loaded onto a Zorbax 300SB-C18 trap column (Agilent, Palo Alto, CA, USA) at a flow rate of 6 μL/min for 10 min, and then separated on a reversed-phase BetaBasic C18 PicoFrit analytical column (0.075 × 250 mm, New Objective, Woburn, MA, USA) using a 90-min linear gradient of 5%–35% acetonitrile in 0.1% formic acid at a flow rate of 250 nL/min. Eluted peptides were sprayed into the Orbitrap Elite equipped with a nano-spray ionization source. Both survey (MS) and product (MS/MS) spectra were acquired in the Orbitrap, and the FTMS resolution was set at 30,000 and 15,000, respectively. Each MS scan was followed by six data-dependent CID MS/MS scans with dynamic exclusion. Other mass spectrometry settings were as follows: spray voltage, 1.5 kV; full MS mass range, *m/z* 300 to 2000; normalized collision energy, 35%. Supplementary Table S1 lists all spectral data of this in-house dataset.

### Pacific Northwest National Laboratory Bacterial Dataset

A public available dataset composed of 53 LCMS experiments for six strains of bacteria was downloaded from the Pacific Northwest National Laboratory (PNNL) website at http://omics.pnl.gov/. This large dataset contains multiple high resolution MS/MS runs per strain. Supplementary Table S2 provides a summary of the dataset downloaded. This dataset was used to gauge the feasibility of the proposed method in performing microbial identification at genus, species, and strain level. Experimental details and optimized sample preparations used to generate this dataset can be found in previously described studies [57, 58]. Here, we briefly mention some important experimental steps that differ between the production of the PNNL dataset and that of the in-house dataset. Bacterial cultures used in the PNNL dataset were diluted to OD 600 = 0.1 and allowed for an overnight growth to reach OD 600 = 3.0. Overnight bacterial cultures were back-diluted to OD 600 = 0.1 and grew in two different flasks at 26°C. The cultures were allowed to grow until OD 600 reached 0.5, at which time one of the flasks was moved to 37°C. Aliquots from both cultures were taken at 0, 1, 2, 4, 8 h and were pooled together into a single flask. For each microbial sample, a modified bead beating method was applied to break the cell walls. Traditional bead beating methods (used to lyse prokaryotes) can produce heated aerosols of the pathogens because of the high speed of shaking. Therefore a vortexing step with beads in solution was used instead and followed by chilling to precipitate aerosols. Trypsin 1:50 (enzyme:protein) ratio was added and digestion occurred at 37°C for 3 h, and the sample was then quickly frozen to stop the digestion.

### Microbial Peptide Sequence Database Construction

A bacterial peptide sequence database was constructed by downloading all bacterial protein sequence fasta files from the National Center for Biotechnology Information (NCBI) at ftp://ftp.ncbi.nlm.nih.gov/genomes/Bacteria on July 15, 2013. A total of 7989010 protein sequences from 2544 strains of bacteria were downloaded and used for database construction. Proteins were in silico digested following the digestion rule for trypsin (i.e., cleaving at the carboxyl terminal of arginine and lysine), allowing up to five missed cleavage sites. In our bacterial peptide sequence database, only *nonredundant* tryptic peptides with molecular weights between 660 and 6000 Da were kept; for each peptide, the names of strains, species, and genera that contain this peptide are also recorded.

**Figure 1:**
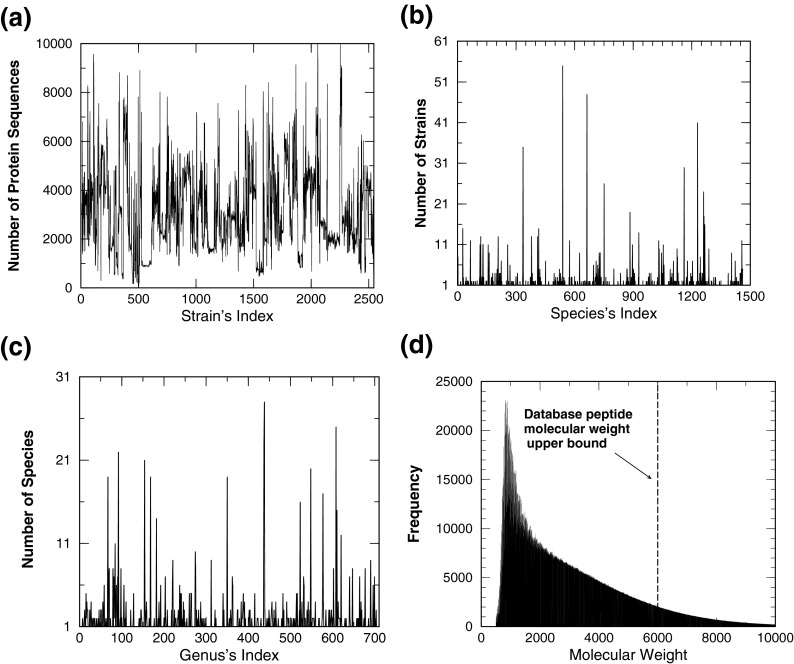
Status of bacterial database used in this investigation. The curve in panel **(a)** shows the number of proteins used for each strain included in the database. Panel **(b)** (**(c)**) displays the number of strains (species) that belongs to a given species (genus). With 0.01 Da as the bin size, panel **(d)** displays the histogram for the number of peptides present in the database as a function of the molecular weight. The vertical dash-line (at 6000 Da) in panel **(d)** indicates the upper bound of molecular weight of peptides included in the current database

Taxonomic information was extracted from the taxonomy files at http://www.ncbi.nlm.nih.gov/Taxonomy/Browser/wwwtax.cgi?name=Archaea&lvl=100 and at http://www.ncbi.nlm.nih.gov/Taxonomy/Browser/wwwtax.cgi?id=2&lvl=100 on July 15, 2013. In the taxonomic files downloaded, some microorganisms were classified only at the genus level but not at the species and the strain level. For these microorganisms, their genera names were also used as their species and strain names. The 2544 bacteria strains downloaded belong to 1461 species and to 706 genera. Panels a, b, and c of Figure 1 display, respectively, the number of protein sequences of each strain, the number of strains belonging to a given species, and the number of species associated with a given genus.

As recommended by previous studies, we employed a decoy database to assess the accuracy of the computed statistical significance [55, 59]. The decoy peptide database was obtained by first reversing the protein sequences followed by the peptide database construction method mentioned above. This database was used for evaluating the accuracy of the spectrum-specific *E*-values assigned to identified peptides and the accuracy of the *E*_*u*_’s assigned to identified microorganisms.

A microbial peptide sequence database could also be constructed using the microorganisms’ genomes. Gene finding tools such as GLIMMER [60, 61] can identify possible genes, which can then be translated into putative proteins [44]. This approach was not pursued here because the microorganisms used in this study have a significant number of proteins available, averaging 3140 protein sequences per strain. However, this approach could be useful for incorporating into the database newly discovered microorganisms whose documented database proteins are few but whose complete genomes are available.

### Software and Parameters Used

Although there exist several software packages that have fully automated peptide identifications [54, 62, 63] and protein identifications [64–66] using MS/MS data, not many have fully automated microbial identifications. MiCId, our pipeline, was designed to fully automate the process, from microbial peptide database construction to microbial identification. The peptide identification component of MiCId is derived from RAId_DbS [54]. The structure and construction of MiCId’s peptide database were described in the previous subsection. In this subsection and the next, we provide the parameters used and detail on how statistical significances are computed for identified microorganisms.

**Figure 2:**
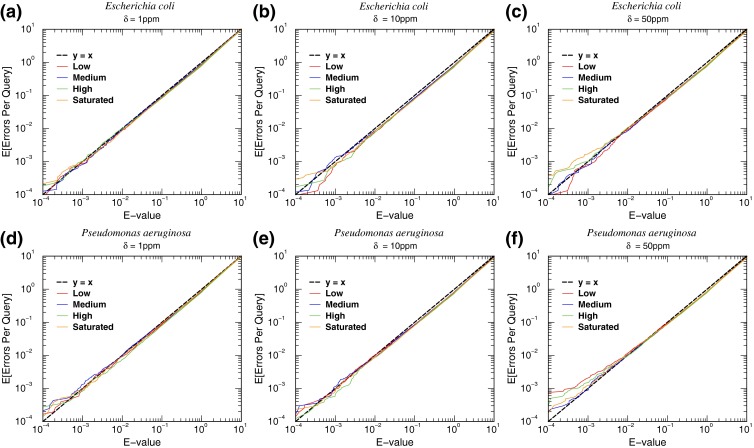
Accuracy assessment of the spectrum-specific *E*-value. The agreement between the expected error per query and the computed *E*-value is examined using the MS/MS spectra from sample numbers 1–4, panels **(a)**–**(c)**, and from sample numbers 5–8, panels **(d)**–**(e)**. The molecular weight (MW) range considered while searching the database is [MW - 3⋅ ***δ*** ⋅MW, MW + 3⋅ ***δ*** ⋅MW]. In each panel the dashed line, ***y*** = ***x***, corresponds to the theoretical line and is used to provide a visual guide regarding how close/off the computed *E*-value curves are from the theoretical line

**Figure 3:**
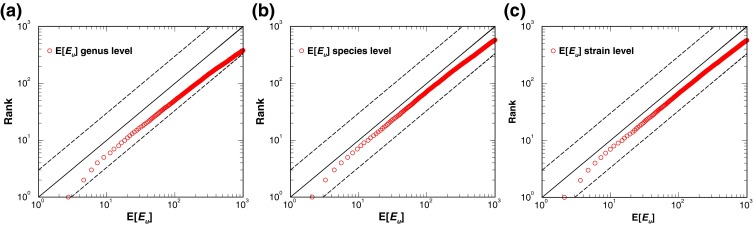
Accuracy assessment of the unified *E*-value (*E*_*u*_). The accuracy of the computed *E*_*u*_s is evaluated by plotting the E[*E*_*u*_] versus rank. The E[*E*_*u*_]s at a given rank were obtained by averaging over all the computed *E*_*u*_s from sample numbers 1–81 having the same rank. The curve made of red circles displayed in panels **(a)**, **(b)**, **(c)** are the curves of E[*E*_*u*_]s for microbial identification performed at the genus, species, and strain level. In each panel, the ***y*** = ***x*** line, corresponding to the theoretical line, together with the two dashed lines, ***y*** = 3***x*** and ***y*** = ***x***/3, provide a visual guide regarding the accuracy of the computed E[*E*_*u*_] curves

The MS/MS spectra used were acquired from iodoacetamide alkylated samples, which were further digested by trypsin. In addition to these conditions, all spectra analyzed had in common the following database search parameters: b and y ions were used for scoring peptides and only peptides with *E*-value less than 10 were kept. To assess the accuracy of the spectrum-specific *E*-values computed for identified peptides, we use spectra from sample number (SN) 1–8 to query a decoy bacterial peptide database with the following parameters: allowing up to five missed cleavage sites (MCS) per peptide and molecular weight error tolerance (MWET) of 1, 5, and 10 ppm for both precursor and product ions (see Figure 2). For statistical accuracy assessment of *E*_*u*_’s computed for identified microorganisms, spectra from SN1–SN81 were used to search a decoy bacterial peptide database with the following parameters: allowing up to five MCS per peptide and MWET of 10 ppm for both precursor and product ions (see Figure 3). To investigate the digestion efficiency of trypsin under protocols one and two, MS/MS spectra from SN1–SN20 were used to search a bacterial peptide database with the following search parameters: maximum of 2, 3, 4, and 5 MCS were allowed per peptide and MWET of 10 ppm for both precursor and product ions (see Table 6). To evaluate the performance of MiCId in microbial identifications, we used spectra from SN1–SN81 to search a bacterial peptide database with the following parameters: allowing up to 5 MCS per peptide and MWET of 10 ppm for both precursor and product ions (see Tables 3, 4, 5, 7, and 8).

**Table 3:** Bacterial Identification at the Genus Level for the PNNL Dataset^a^

*Escherichia coli K-12* sample number 29-39							
Genus	IF	E[R]	E[ln (*E*_*u*_)]	E[WPC]	E[NIP]	E[NUP]	E[CI]
*Escherichia*	11/11	1.0	–6498.6 ± 349.0	597.7	1594	15	1
*Halorhodospira*	2/11	2.0	–14.4 ± 3.0	1.7	12	1	6
*C. Puniceispirillum*	1/11	2.0	–6.4 ± 0.0	1.0	3	1	38
*Enterococcus*	1/11	3.0	–4.4 ± 0.0	1.0	2	1	5
*Lacinutrix*	1/11	2.0	–3.9 ± 0.0	1.0	2	0	24
*Mycobacterium tuberculosis H37Rv* sample number 40-48							
Genus	IF	E[R]	E[ln (*E*_*u*_)]	E[WPC]	E[NIP]	E[NUP]	E[CI]
*Mycobacterium*	9/9	1.0	–6784.8 ± 729.6	725.6	937	433	1
*Ethanoligenens*	1/9	2.0	–6.3 ± 0.0	1.0	1	1	65
*Salmonella*	3/9	2.3	–5.6 ± 0.3	1.0	1	1	15
*Methanoplanus*	1/9	2.0	–4.5 ± 0.0	1.0	1	0	316
*Treponema*	2/9	2.5	–2.9 ± 0.1	0.5	3	0	3
*Salmonella typhimurium ATCC 14028* sample number 49-56							
Genus	IF	E[R]	E[ln (*E*_*u*_)]	E[WPC]	E[NIP]	E[NUP]	E[CI]
*Salmonella*	8/8	1.0	–5596.0 ± 670.5	546.8	1050	204	1
*Halorhodospira*	1/8	2.0	–14.4 ± 0.0	2.0	6	1	10
*Planctomyces*	1/8	2.0	–6.7 ± 0.0	1.0	2	1	14
*Mycoplasma*	1/8	3.0	–2.1 ± 0.0	1.0	5	0	2
*Gordonia*	3/8	5.3	–0.8 ± 2.4	0.7	3	0	7
*Yersinia pestis CO92* sample number 57-65							
Genus	IF	E[R]	E[ln (*E*_*u*_)]	E[WPC]	E[NIP]	E[NUP]	E[CI]
*Yersinia*	9/9	1.0	-9201.2 ± 897.8	847.0	1336	466	1
*Azospirillum*	1/9	2.0	–6.8 ± 0.0	1.2	6	0	2
*C. Carsonella*	1/9	2.0	–5.1 ± 0.0	1.0	1	1	9
*Tannerella*	1/9	3.0	–4.1 ± 0.0	1.0	3	0	16
*Novosphingobium*	1/9	3.0	–4.1 ± 0.0	1.2	7	0	10
*Yersinia pseudotuberculosis PB1 Plus* sample number 66-74							
Genus	IF	E[R]	E[ln (*E*_*u*_)]	E[WPC]	E[NIP]	E[NUP]	E[CI]
*Yersinia*	9/9	1.0	–8043.2 ± 803.2	749.7	1187	400	1
*Novosphingobium*	1/9	2.0	–21.3 ± 0.0	3.0	9	2	7
*Syntrophus*	4/9	3.5	–1.5 ± 4.3	0.9	5	0	11
*C. Uzinura*	4/9	2.8	0.1 ± 0.8	0.2	2	0	63
*Arcobacter*	2/9	4.5	0.4 ± 0.4	0.5	4	0	5
*Shewanella oneidensis MR-1* sample number 75-81							
Genus	IF	E[R]	E[ln (*E*_*u*_)]	E[WPC]	E[NIP]	E[NUP]	E[CI]
*Shewanella*	7/7	1.0	–14534.8 ± 8196.6	1369.1	1841	1022	1
*Cupriavidus*	1/7	2.0	–15.5 ± 0.0	3.2	15	1	7
*Kyrpidia*	1/7	2.0	–7.7 ± 0.0	1.0	1	1	49
*Azoarcus*	1/7	3.0	–5.6 ± 0.0	1.8	19	0	6
*Polaromonas*	1/7	3.0	–4.1 ± 0.0	0.7	6	0	12

The numerical entries in the table are the expected values E[X]. The E[ln (*E*_*u*_)] is followed by its standard deviation ± *σ*_*X*_

**Table 4:** Bacterial Identification at the Species Level for the PNNL Dataset

*Escherichia coli K-12* sample number 29-39							
Species	IF	E[R]	E[ln (*E*_*u*_)]	E[WPC]	E[NIP]	E[NUP]	E[CI]
*E. coli*	11/11	1.0	–6473.8 ± 341.9	595.4	1582	14	1
*H. halophila*	2/11	2.0	–14.0 ± 3.3	1.7	12	1	2
*C.P. marinum*	1/11	2.0	–6.2 ± 0.0	1.0	3	1	50
*E. hirae*	1/11	3.0	–5.7 ± 0.0	1.0	2	1	27
*Ruminococcus*	1/11	3.0	–3.7 ± 0.0	1.0	2	1	31
*Mycobacterium tuberculosis H37Rv* sample number 40-48							
Species	IF	E[R]	E[ln (*E*_*u*_)]	E[WPC]	E[NIP]	E[NUP]	E[CI]
*M. tuberculosis*	9/9	1.0	–3697.0 ± 394.8	391.5	887	5	1
*E. harbinense*	1/9	2.0	–5.7 ± 0.0	1.0	1	1	93
*M. petrolearius*	1/9	2.0	–3.7 ± 0.0	1.0	1	0	550
*P. oguniense*	1/9	2.0	–3.6 ± 0.0	1.5	2	0	779
*S. smaragdinae*	1/9	3.0	–2.7 ± 0.0	1.0	1	0	47
*Salmonella typhimurium ATCC 14028* sample number 49-56							
Species	IF	E[R]	E[ln (*E*_*u*_)]	E[WPC]	E[NIP]	E[NUP]	E[CI]
*S. enterica*	8/8	1.0	–5099.6 ± 604.7	498.5	1043	133	1
*H. halophila*	1/8	2.0	–14.3 ± 0.0	2.0	6	1	4
*P. limnophilus*	1/8	2.0	–6.9 ± 0.0	1.0	2	1	24
*D. salexigens*	1/8	2.0	–5.7 ± 0.0	1.2	3	1	13
*C.M. haemolamae*	1/8	3.0	–5.6 ± 0.0	1.0	1	0	220
*Yersinia pestis CO92* sample number 57-65							
Species	IF	E[R]	E[ln (*E*_*u*_)]	E[WPC]	E[NIP]	E[NUP]	E[CI]
*Y. pestis*	9/9	1.0	–5888.8 ± 566.1	549.4	1317	24	1
*N. PP1Y*	1/9	4.0	–7.0 ± 0.0	1.5	7	0	4
*C.C. ruddii*	1/9	2.0	–4.5 ± 0.0	1.0	1	1	3
*T. forsythia*	1/9	3.0	–3.8 ± 0.0	1.0	3	0	11
*Ruminococcus*	1/9	3.0	–3.7 ± 0.0	1.0	2	0	43
*Yersinia pseudotuberculosis PB1 Plus* sample number 66-73							
Species	IF	E[R]	E[ln (*E*_*u*_)]	E[WPC]	E[NIP]	E[NUP]	E[CI]
*Y. pseudotuberculosis*	9/9	1.0	–5198.7 ± 516.8	490.1	1173	23	1
*N. aromaticivorans*	1/9	2.0	–15.9 ± 0.0	2.5	6	2	4
*S. aciditrophicus*	4/9	4.2	–0.3 ± 4.5	0.8	5	0	4
*C. U. diaspidicola*	4/9	2.2	0.6 ± 0.9	0.2	2	0	116
*M. ruber*	1/9	2.0	0.8 ± 0.0	0.5	3	0	21
*Shewanella oneidensis MR-1* sample number 74-81							
Species	IF	E[R]	E[ln (*E*_*u*_)]	E[WPC]	E[NIP]	E[NUP]	E[CI]
*S. oneidensis*	7/7	1.0	–10280.9 ± 5954.4	949.7	1660	402	1
*K. tusciae*	1/7	2.0	–7.6 ± 0.0	1.0	1	1	35
*A. BH72*	1/7	2.0	–5.2 ± 0.0	1.7	11	0	7
*P. NH8B*	1/7	5.0	–3.8 ± 0.0	1.8	11	0	6
*M. versatilis*	1/7	2.0	–3.7 ± 0.0	1.0	3	1	20

The numerical entries in the table are the expected values E[X]. The E[ln (*E*_*u*_)] is followed by its standard deviation ± *σ*_*X*_

**Table 5:** Bacterial Identification at the Strain Level for the PNNL Dataset^a^

Strain	IF	E[R]	E[ln (*E*_*u*_)]	E[WPC]	E[NIP]	E[NUP]	E[CI]
*E.c. K-12 MG1655*	11/11	1.1	–6363.9 ± 333.9	583.0	1566	0	1
*E.c. K-12 W3110*	11/11	2.0	–6353.7 ± 331.3	582.0	1564	0	1
*E.c. BW2952*	11/11	2.9	–6339.0 ± 332.1	580.9	1561	0	1
*H.h. SL1*	2/11	4.0	–13.9 ± 3.4	1.7	12	1	2
*C.P. m.IMCC1322*	1/11	4.0	–6.1 ± 0.0	1.0	3	1	66
*Mycobacterium tuberculosis H37Rv* sample number 40-48							
Strain	IF	E[R]	E[ln (*E*_*u*_)]	E[WPC]	E[NIP]	E[NUP]	E[CI]
*M.t. H37Rv*	9/9	1.3	–3656.0 ± 386.2	387.3	884	0	1
*M.t. H37Ra*	9/9	1.7	–3652.6 ± 386.4	386.9	883	0	1
*M.t. F11*	9/9	3.9	–3640.2 ± 384.6	385.9	881	0	1
*M.t. KZN4207*	9/9	4.2	–3639.3 ± 383.3	385.8	880	0	1
*M.t. CTRI2*	9/9	4.6	–3637.0 ± 381.9	385.5	880	0	1
*Salmonella typhimurium ATCC 14028* sample number 49-56							
Strain	IF	E[R]	E[ln (*E*_*u*_)]	E[WPC]	E[NIP]	E[NUP]	E[CI]
*S.T. 14028S*	8/8	1.9	–4093.9 ± 498.5	403.9	1027	0	1
*S.T. UK1*	8/8	2.5	–4089.1 ± 493.6	403.3	1025	0	1
*S.T. T000240*	8/8	3.0	–4087.1 ± 495.2	403.0	1025	0	1
*S.T. U288*	8/8	4.1	–4083.3 ± 497.5	402.5	1024	0	1
*S.T. ST4-74*	8/8	4.1	–4080.9 ± 494.9	402.6	1023	0	1
*Yersinia pestis CO92* sample number 57-65							
Strain	IF	E[R]	E[ln (*E*_*u*_)]	E[WPC]	E[NIP]	E[NUP]	E[CI]
*Y.p. CO92*	9/9	1.0	–5699.2 ± 545.9	531.8	1317	0	1
*Y.p. M. 91001*	6/9	2.2	–5452.2 ± 346.2	512.0	1266	0	1
*Y.p. KIM10*	3/9	3.3	–5272.1 ± 273.3	497.7	1231	0	1
*Y.p. P. F*	1/9	2.0	–5071.8 ± 0.0	486.0	1212	0	1
*N. PP1Y*	1/9	4.0	–6.9 ± 0.0	1.5	7	0	4
*Yersinia pseudotuberculosis PB1 Plus* sample number 66-74							
Strain	IF	E[R]	E[ln (*E*_*u*_)]	E[WPC]	E[NIP]	E[NUP]	E[CI]
*Y.p. PB1*	9/9	1.0	–5048.1 ± 502.3	475.7	1172	3	1
*Y.p. IP32953*	7/9	2.0	–4868.1 ± 499.6	463.8	1145	0	1
*N.a. DSM12444*	1/9	3.0	–15.7 ± 0.0	2.5	6	2	4
*S.a. SB*	4/9	7.8	–0.1 ± 4.5	0.8	5	0	4
*C.U. d. ASNER*	4/9	2.8	0.7 ± 1.0	0.2	2	0	184
*Shewanella oneidensis MR-1* sample number 75-81							
Strain	IF	E[R]	E[ln (*E*_*u*_)]	E[WPC]	E[NIP]	E[NUP]	E[CI]
*S.o. MR1*	7/7	1.0	–10280.4 ± 5954.4	949.5	1660	402	1
*K.t. DSM2912*	1/7	2.0	–7.6 ± 0.0	1.0	1	1	54
*C.m. CH34*	1/7	3.0	–6.4 ± 0.0	1.1	8	0	2
*A. BH72*	1/7	2.0	–4.2 ± 0.0	1.7	11	0	4
*M. 301*	1/7	2.0	–3.7 ± 0.0	1.0	3	1	13

The numerical entries in the table are the expected values E[X]. The E[ln (*E*_*u*_)] is followed by its standard deviation ± *σ*_*X*_

The computational time required for running MiCId depends on the computational resources employed. As an illustration, the microbial peptide database (needed for this study) construction takes about 3 h using a 2.4 GHz logical core and occupies 80 GB of disk space. However, it is important to note that this database construction is a one-time event; the constructed database can be used for all subsequent spectral analyses. In terms of microbial identification via spectral analyses, for a dataset composed of 18,000 MS/MS spectra, it takes MiCId about 15 min to finish the analyses using 4 2.4GHz logical cores in parallel. The computational/analysis time can be reduced by increasing the number of logical cores used.

### Statistical Method for Microbial Identification

The efficacy of our statistical method relies on two assumptions: (1) statistical significances, *E*-values (*P*-values), assigned at the peptide level are accurate; (2) microorganisms used for database construction are correctly classified into the hierarchy of strains, species, and genera. The first assumption can be verified computationally by searching a database made of decoy/random peptides [55] with a set of MS/MS spectra as queries. A decoy database was created using the same procedure employed to generate a microbial peptide sequence database except that each downloaded protein sequence had its sequence reversed. MS/MS spectra of SN1-SN8 were used as queries to search the decoy database and the expected number of errors per query below an *E*-value cutoff was computed and plotted versus the cutoff *E*-value. Figure 2 shows that the computed curves trace very closely the theoretical line, *y* = *x*, indicating that the computed *E*-values assigned to peptides identified are indeed accurate. Regarding the second assumption, microorganisms’ taxonomic classification has improved and is expected to continually improve because of advances in DNA sequencing technology and a polyphasic approach that utilizes genotypic, chemotypic, and phenotypic information during taxonomic classification [67]. However, taxonomic classification of some microorganisms appears dubious at the moment and could influence microorganism identification [50]. For example, some studies suggest that the *Shigella flexneri* species should be classified as a strain of *Escherichia coli* [68, 69].

To provide statistical significance at the genus, species, and strain levels, one may compute a unified *E*-value *E*_*u*_ by combining the spectrum-specific *E*-values of the CIPs belonging to the same genus, species, and strain, respectively. The spectrum-specific *E*-value assigned to a peptide is given by multiplying the database size *n*_*mw*_ (Bonferroni’s correction factor) by that peptide’s spectrum-specific *P*-value (*P*), i.e.,

1 $$ E={n}_{mw}\times P, $$

where *n*_*mw*_ is the total number of qualified peptides in the database (i.e., peptides that are within the allowed precursor ion MWET).

We compute *E*_*u*_, the unified *E*-value, by executing the following steps. First, we obtain a corresponding set of CIPs identified with an appropriate *E*-value cutoff; second, based on the CIP coverage, we cluster at either genus, species, or strain level; third, appropriate weights are then assigned to each CIP; finally, the unified *P*-value is computed, yielding the unified *E*-value upon multiplication of the correct Bonferroni factor.

The first step is important. For a given LCMS experiment, thousands of MS/MS spectra are analyzed and by random chance some identified peptides will have statistically significant *E*-values (SSEs). Identified peptides with SSEs that occur by chance are spurious and are called FP. In this study, for each sample analyzed, only peptides with *E*-values less than or equal to

$$ {E}_c\equiv 1/{n}_s, $$

where *n*_*s*_ is the total number of MS/MS spectra of the sample, are used to compute the *E*_*u*_.

When a peptide is identified in multiple spectral searches with *E*-value less than *E*_*c*_, the smallest *E*-value of the identified peptide is kept and the rest of the *E*-values are ignored. We choose the most conservative approach by keeping only the smallest *E*-value and we have not explored the possibility of combining *E*-values corresponding to the same peptide.

We then transform the *E*-values of CIPs into the database *P*-values. This is accomplished by assuming that the occurrence of peptides with SSEs (i.e., peptides with small *E*-values) in a database are infrequent events and can be modelled by a Poisson process [59, 70]. The *E*-values are then transformed into the database *P*-values by

2 $$ P(E)=1-{e}^{-E}, $$

where *P(E)* represents the probability that at least one event occurs by random chance with *E*-value less than or equals to *E*.

The second step is necessary since microorganisms can have highly homologous proteomes. As a consequence, a large number of CIPs may be shared among many microorganisms. To avoid false identification, it is necessary to have a clustering procedure to group microorganisms that share a significant number of CIPs.

We employ a peptide-driven clustering procedure whose algorithm is described below. First, CIPs are assigned to the different genera of microorganisms present in the database. Second, genera are sorted in order of decreasing number of CIPs. Third, starting from the best ranked genus, any other lower ranked genus will cluster to the former if 7/8 or more of the latter’s identified peptides are also identified by the former. Once the worst ranked genus is reached, the process will repeat itself until all the unclustered genera have been used as a starting point, but not more than once. The first genus entering a cluster is called the head of that cluster, whereas other genera are the members of that cluster. Each cluster is assigned a cluster index (CI), which is shared by the head and the members of that cluster. There is, however, an exception to the general clustering rule. When a genus contains five or more evidence peptides that are not shared with other genera, it can only be a cluster head. Each cluster head is then selected as the best representative genus for its cluster.

The identical clustering procedure is used for identifications at species and strain levels. Our clustering procedure assumes that in the database the correct microorganisms are present and their proteomes should explain the majority of the CIPs. If the correct microorganisms are not in the database, the closely related ones should explain the majority of CIPs. Therefore, MiCId provides microbial identifications if we know a priori that the underlying microorganism is in the database; on the other hand, if we know a priori that the underlying microorganism is not in the database, MiCId can be used for microbial classifications.

The third step is to weight the database *P*-value of an identified peptide *π* according to the number of genus clusters (*C*_*π*,*g*_), species clusters (*C*_*π*,*s*_), or strain clusters (*C*_*π*,*ss*_) that contain *π* in their proteomes. Specifically, when conducting identification at the genus, species, or strain level, *π*’s database *P*-value is adjusted by raising it to the power of *w*_*π*_, where *w*_*π*_ is define as 1/(*C*_*π*,*g*_ !), 1/(*C*_*π*,*s*_ !), or 1/(*C*_*π*,*ss*_ !), respectively. Note that 1/*C* ! is the simplex volume bounded by *x*_1 ≤ *i* ≤ *C*_ ≥ 0 and ${\displaystyle {\sum}_{i=1}^C}{x}_i\le 1$. This procedure is applied to all peptides identified with *E*-value less than 1, not just to CIPs. Apparently, incorrect taxonomic classification or missing polymorphism information might reduce the value of *C*_*π*,*g*_, *C*_*π*,*s*_,and *C*_*π*,*ss*_ used to adjust *π*’s database *P*-value, yielding a stronger weight than warranted. To prevent a CIP from having excessive weight, we shall use the value 1/2 for both *C* = 1 and *C* = 2.

Finally, to obtain an unified *P*-value (*P*_*u*_), let $\left\{{\widehat{p}}_1={p}_1^{w_1},{\widehat{p}}_2={p}_2^{w_2},\dots, {\widehat{p}}_{n_g}={p}_{n_g}^{w_{n_g}}\right\}$ be the set of adjusted *P*-values of CIPs belonging to a given genus *g*. The same procedure, with *n*_*g*_ → *n*_*s*_ (or *n*_*ss*_), can be used at species and strain levels. The ${\hat{p}}_i$’s are then combined into a new variable

3 $$ \tau =\prod_{i=1}^{n_g}{\widehat{p}}_i, $$

which is compared with the stochastic variable

$$ \tilde{\tau}={x}_m^{\left({m}_{\mathrm{raw}}+1-m\right)}{\displaystyle \prod_{j=1}^{m-1}}{x}_j, $$

where the *x*_*j*_s are independently uniformly distributed random variables in the range [0, 1), ${m}_{\mathrm{raw}}\equiv {\displaystyle {\sum}_{i=1}^{n_g}}{w}_i\left(E\le {E}_c\right)$ is the effective number of independent *P*-values, and *m*≡⌈*m*_raw_⌉ is the smallest integer that is greater than or equal to *m*_raw_. Then a unified conditional probability is computed by extending the formula for the product of truncated *P*-values [71]

$$ {Z}_t\left(\left.{\displaystyle \prod_{j=1}^m}{x}_j\le \tau \right|\ m\right)=\frac{\tau }{P_c^m}{\displaystyle \sum_{s=0}^{m-1}}\frac{{\left[m \ln\ \left({P}_c\right)- \ln\ \left(\tau \right)\right]}^s}{s!} $$

to

4 $$ {P}_t\left(\tilde{\tau}\le \tau \Big|m,{m}_{\mathrm{raw}}\right)=\frac{\tau }{P_c^{m_{raw}}}{\displaystyle \sum_{s=0}^{m-1}}\frac{{\left[{m}_{raw} \ln\ \left({P}_c\right)- \ln\ \left(\tau \right)\right]}^s}{s!}. $$

In Equation 4, *P*_*c*_ ≡ *P*(*E*_*c*_);

there is a reason that *m*_raw_ is not rounded to *m* in the expression $\tau /{P}_c^{m_{\mathrm{raw}}}$ in Equation 4. Each *P*-value, before being weighted and combined, has to be less than *P*_*c*_. That is,

5 $$ \tau ={\displaystyle \prod_i}{p}_i^{w_i}\le {\displaystyle \prod_i}{P}_c^{w_i}={P}_c^{m_{\mathrm{raw}}}. $$

If one blindly rounds up *m*_raw_, say from *m*_raw_ = 0.01 to 1, it is very likely to have *τ* > *P*_*c*_^(*m* = 1)^, violating the fundamental inequality (5). With the conditional probability for the product of truncated *P*-values given, we can write down the unified *P*-value as

6 $$ \begin{array}{c}\hfill {P}_u\left(\tilde{\tau}\le \tau \right)=\frac{M!}{m!\left(M-m\right)!}{P}_c^m{\left(1-{P}_c\right)}^{M-m}{P}_t\left(\tilde{\tau}\le \tau \Big|\ m,{m}_{\mathrm{raw}}\right)\hfill \\ {}\hfill +{\displaystyle \sum_{j=m+1}^M}\kern0.5em \frac{M!}{j!\left(M-j\right)!}{P}_c^j{\left(1-{P}_c\right)}^{M-j}\times \hfill \\ {}\hfill \times \left[\theta \left({P}_c^j-\tau \right){P}_t\left(\tilde{\tau}\le \tau \Big|j,j\right)+\theta \left(\tau -{P}_c^j\right)\right], \hfill \end{array} $$

where $M=\left\lceil {\displaystyle {\sum}_{j=1}^{N_g}}{w}_j\left(E\le 1\right)\right\rceil$ with *N*_*g*_ being the total number of identified peptides (with *E*-value ≤ 1) mappable to genus *g*, and *θ*(*x*) takes value 1 when *x* > 0 and 0 otherwise. The first term of the right hand side of Equation 6 contains the conditional probability (4) and a binomial factor that gives the probability of getting *m* peptides (each with *P*-value less than *P*_*c*_) out of *M* peptides. Consider sample *M* independently uniformly distributed random numbers in the range[0, 1]).

The product of the two aforementioned contributions, namely, the binomial factor and the truncated *P*-value (4), gives the joint probability for obtaining *m* random numbers, each less than *P*_*c*_, whose product is less than *τ*. Each additional term in Equation 6 carries a similar meaning: it represents the joint probability, when sampling *M* random numbers, for obtaining *j* random numbers, each less than*P*_*c*_, whose product is less than *τ*. The unified *P*-value is computed for an example in the electronic supplementary material.

The unified *E*-value *E*_*u*_ is then obtained by

7 $$ {E}_u={P}_u\times \mathrm{B}\left(E\le {E}_c\right), $$

where the Bonferroni correction factor *B*(*E* ≤ *E*_*c*_) denotes the number of genus clusters, species clusters, or strain clusters that contain at least one evidence peptide with *E*-value less than the cutoff *E*_*c*_ = 1/*n*_*s*_.

## Results

We have mentioned, in the Introduction, that using a spectrum-specific significance measure (such as *E*-value) enables comparison/unification of statistical significances. Evidently, the unified significance measure can be accurate only if the per-spectrum significance assignments are accurate and the method to combine them is rigorous. There is no doubt that the accuracy of the unified *E*-value critically affects the performance of our application.

To evaluate the accuracy of the computed *E*_*u*_, we used spectra from SN1–SN81 to query a decoy bacterial peptide database, whose construction was described earlier. Panels a, b, and c of Figure 3 display the curves of the expected *E*_*u*_ (E[*E*_*u*_]) versus rank for microbial identifications at the genus, species, and strain levels respectively. E[*E*_*u*_] at a given rank was computed by averaging over all the *E*_*u*_ s of identified microorganisms having that rank from results of SN1 through SN81. Identified microorganisms are ranked by *E*_*u*_ in ascending order, meaning the best ranked microorganism has the smallest *E*_*u*_. If the computed *E*_*u*_s are accurate, plotting E[*E*_*u*_] versus its corresponding rank should yield a curve close to the *y* = *x* line. As shown in Figure 3, these curves are bounded by the two dotted lines, *y* = 3*x* and *y* = *x*/3, indicating that on average the computed *E*_*u*_s are no more than a factor of three off. Further, these curves seem always to lie below the *y* = *x* line, suggesting that the computed *E*_*u*_s are conservative.

The PNNL and the in-house datasets were used to evaluate MiCId’s microbial identification. Within either dataset, we average the analysis results from samples containing the same underlying organism. A microorganism *o* (not necessarily the underlying organism of the samples) may be reported in analyses of *A* out of *B* samples. In this case, the microorganism *o* is said to have identification fraction (IF) equal to *A*/*B*. For microorganism *o*, the expected values (averaged only over the *A* samples that report *o*) of rank (R), natural log of *E*_*u*_, weighted peptide count (WPC), the number of identified peptides (NIP), the number of unique peptides (NUP), and cluster index (CI) are computed and denoted, respectively, by E[R], E[ln *E*_*u*_], E[WPC], E[NIP], E[NUP], and E[CI]. To be more precise, for each sample analyzed, R is the rank of *o* in the identified microorganisms when sorted in increasing order of assigned *E*_*u*_, computed via Equation 7; WPC is defined as the sum of weights (*w*_*i*_) of identified peptides mappable to *o*; NIP is the number of identified peptides belonging to *o*; NUP is the number of identified peptides belonging *exclusively* to *o*; CI is the index for the cluster *o* belongs to.

### Microbial Identification for PNNL Dataset

To evaluate the effectiveness of MiCId in terms of microbial identification at the genus, species, and strain levels, we run MiCId using spectra from SN29 through SN81 (the whole PNNL dataset). Bacterial identification at genus level for the PNNL dataset is displayed in Table 3, within which one sees that for each sample the correct genus is identified and ranked number one (E[R] = 1). Table 3 also shows that the correct genera identified have E[NUP] greater than zero and also greater than the E[NUP] of the lower-ranked genera.

Table 4 shows that the correct species are identified with rank one for all the PNNL’s samples. It also shows that the correct species’s E[NUP] remains positive but smaller than the corresponding genus’s E[NUP]. This is expected because the proteome (or peptidome) similarity among species within the same genus tends to be stronger than that among genera. Table 4 also shows that the clustering procedure manages to cluster identified species that are statistically significant and sharing identical peptides, thus preventing false identifications. In Table 4, in terms of E[NIP] and E[WPC], we also noted a clear separation between the best ranked species and the lower-ranked species.

Table 5 shows the results for microbial identification at the strain level. Consistently correct identification across all samples at the strain level was obtained only for three strains: *Yersinia pestis CO92*, *Yersinia pseudotuberculosis PB1*, and *Shewanella oneidensis MR-1*. The other three strains, *Escherichia coli K-12*, *Mycobacterium tuberculosis H37Rv*, and *Salmonella typhimurium ATCC 14028* were identified with expected ranks better than two. The total number of strains present in the database varies by species: 58 *Escherichia coli* strains, 29 *Salmonella typhimurium* strains, 19 *Mycobacterium tuberculosis* strains, 12 *Yersinia pestis* strains, 4 *Yersinia pseudotuberculosis* strains, and 1 *Shewanella oneidensis* strain. The large number of strains within each of the first three species might partially explain why it was difficult to have the correct strains rank number one across all samples. An in-depth discussion of this difficulty based on peptidome similarities among strains is given in the Discussion section. Although the correct strains were not always identified as the best ranked ones, they were, however, always identified within the best ranking strain cluster. That is, for the correct strains E[CI] = 1 even when E[R]> 1.

### Microbial Identification for the In-House Dataset

The in-house dataset, produced using different sample preparation methods from the PNNL dataset, were used to examine the robustness of the analysis pipeline for microorganism identifications. The main difference between batch one and the other two batches was how the tryptic digestion of protein was carried out. In batches two and three, the protein digestion step of batch one was modified by adding a cleavable surfactant prior to trypsin digestion, aiming to increase the number of CIPs. Table 6 displays, for samples collected at different ODs, the CIP counts as the maximally allowed MCS of candidate peptides increases from two to five. The number of CIPs in batch two appears to be higher than that in batch one. However, due to the limited number of data points and the fact that the samples were acquired at different OD values, the robustness of this trend should be verified by a larger study with more data points collected at same OD values.

**Table 6:** The Number of CIPs at the 1% False Discovery Rate for the In-House Dataset

Sample from batch one					Samples from batch two			
*Escherichia coli*					*Escherichia coli*			
SN	1	2	3	4	9	10	11	12
OD	0.30	0.75	1.07	1.34	0.34	0.66	1.01	1.34
NMCS	Number of CIPs				Number of CIPs			
2	67	129	15	47	480	197	214	486
3	101	186	23	72	731	303	322	725
4	132	227	30	95	941	385	415	906
5	155	264	39	116	1077	456	484	1036
*Pseudomonas aeruginosa*					*Pseudomonas aeruginosa*			
SN	5	6	7	8	13	14	15	16
OD	0.38	0.65	0.90	1.50	0.43	0.64	1.12	1.50
NMCS	Number of CIPs				Number of CIPs			
2	115	147	70	147	187	175	166	177
3	196	383	129	244	320	294	281	296
4	266	514	180	317	420	377	372	389
5	311	610	228	376	491	448	436	456
					*Salmonella enterica*			
SN					17	18	19	20
DO					0.42	0.68	0.96	1.34
NMCS	Number of CIPs				Number of CIPs			
2					186	186	156	179
3					283	284	233	263
4					367	365	291	334
5					427	429	337	378

**Figure 4:**
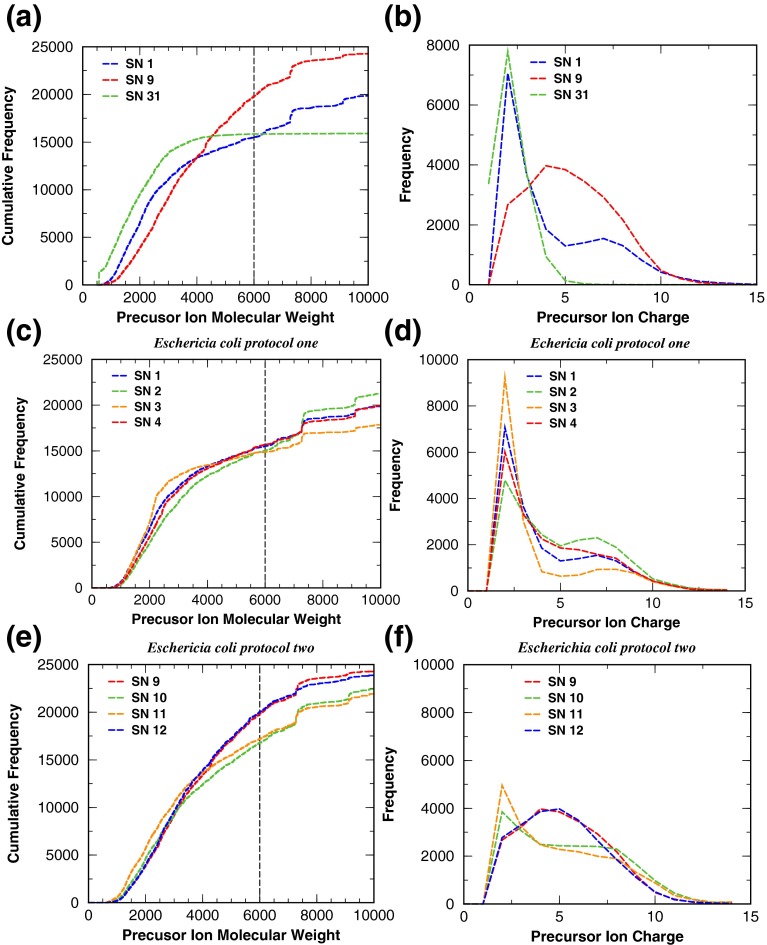
Molecular weight and charge distribution. The curves in panels **(a)**–**(f)** display the molecular weight and charge distributions obtained for the in-house dataset under protocols one and two. Also displayed in panels **(a)** and **(b)** are the molecular weight and charge distributions for SN 31 from the PNNL dataset. Similar molecular weight and charge distributions like the one observed for SN 31 are obtained for the other samples from the PNNL dataset

Figure 4 displays the precursor ions’ molecular weight and charge distributions. The green curves in panels a and b are for SN31 (from the PNNL dataset), and they show that out of the 15,988 MS/MS spectra of SN31, approximately 85% have precursor ion molecular weights less than 3000 Da, and that the average precursor ion charge state is about 3. Similar results are also obtained for the other samples within the PNNL dataset (data not shown). For the in-house dataset, the curves for SN1 (blue) and SN9 (red) show, respectively, that about 55% and 37% of precursor ions have molecular weights less than 3000 Da, a considerable difference from the 85% obtained for the PNNL dataset. This difference is probably due to the different procedures used for sample preparation. Comparing the curves of panel d with those of panel f, one finds that the average precursor ion charge is around 3 for SN1-4, whereas for SN9-12 it is around 5. Panels a, c, and e of Figure 4 show that the tryptic peptides produced by the in-house procedures tend to be longer than those in the PNNL samples. Given that precursor ion charge determination for longer peptides can be inaccurate, this may partly explain the lower number of CIPs obtained from these samples than from the PNNL samples.

Tables 7 and 8 summarize the results obtained for microbial identifications at the genus and species levels for the in-house dataset. Individual analysis for each batch can be found in the Supplementary Tables S3-S8. Tables 7 and 8 show that E[NUP]s for *Pseudomonas aeruginosa* and *Salmonella enterica* samples remain relatively large given that the number of CIPs from the in-house dataset are much smaller than that from the PNNL dataset. As for the *Escherichia coli* samples, a low E[NUP] was observed and the correct genus was not always identified as the top ranking one. This can be due to several reasons. First, it is known that the *Escherichia coli* proteome contains a trypsin inhibitor [72], undermining the digestion efficiency of trypsin, producing less complete digestions, and consequently yielding a low number of CIPs. Second, the closeness between *Shigella* and *Escherichia* [68, 69] robs E[NUP] out of *Escherichia*, lowering the identification confidence of *Escherichia*. The third cause is common to all the in-house samples: our lysis procedure via formic acid might not be as efficient in breaking the cell walls as using mechanical disruptions [58, 73].

**Table 7:** Bacterial Identification at the Genus Level for the In-House Dataset^a^

*Escherichia coli* sample number 1-4, 9-12, and 21-26							
Genus	IF	E[R]	E[ln (*E*_*u*_)]	E[WPC]	E[NIP]	E[NUP]	E[CI]
*Escherichia*	14/14	1.4	–365.2 ± 290.4	42.1	115	1	1
*Shigella*	14/14	1.6	–349.6 ± 271.8	40.1	114	0	1
*Enterobacter*	2/14	5.0	–36.8 ± 24.4	5.1	18	0	1
*Enterobacteriaceae*	1/14	3.0	–13.9 ± 0.0	1.8	8	0	1
*Citrobacter*	1/14	4.0	–13.2 ± 0.0	1.5	6	0	1
*Pseudomonas aeruginosa* sample number 5-8, 13-16 and 27-28							
Genus	IF	E[R]	E[ln (*E*_*u*_)]	E[WPC]	E[NIP]	E[NUP]	E[CI]
*Pseudomonas*	10/10	1.0	–598.7 ± 647.8	71.3	102	34	1
*Acidovorax*	1/10	2.0	–23.0 ± 0.0	2.0	4	2	3
*Azospira*	5/10	4.2	–5.6 ± 8.3	1.2	2	0	3
*Thiobacillus*	1/10	2.0	–5.6 ± 0.0	1.0	2	1	5
*Rothia*	1/10	3.0	–4.8 ± 0.0	1.0	1	0	7
*Salmonella enterica* sample number 17-20							
Genus	IF	E[R]	E[ln (*E*_*u*_)]	E[WPC]	E[NIP]	E[NUP]	E[CI]
*Salmonella*	4/4	1.0	–232.1 ± 21.1	27.1	61	7	1
*Haloferax*	2/4	2.5	–7.2 ± 0.5	1.0	1	1	10
*Pseudovibrio*	1/4	2.0	–5.5 ± 0.0	1.0	1	0	11
*Cupriavidus*	4/4	2.8	–5.3 ± 2.5	1.1	2	1	6
*Aliivibrio*	3/4	4.0	–1.7 ± 2.3	0.8	2	0	4

^a^ The numerical entries in the table are the expected values E[X]. The E[ln (*E*_*u*_)] it is followed by its standard deviation ± *σ*_*X*_

**Table 8:** Bacterial Identification at the Species Level for the In-House Dataset

*Escherichia coli* sample number 1-4, 9-12, and 21-26							
Species	IF	E[R]	E[ln (*E*_*u*_)]	E[WPC]	E[NIP]	E[NUP]	E[CI]
*E. coli*	14/14	1.4	–364.2 ± 288.0	41.9	115	1	1
*S. boydii*	10/14	2.1	–188.3 ± 80.8	22.8	65	0	1
*S. flexneri*	10/14	4.1	–178.0 ± 76.3	22.1	63	0	1
*S. dysenteriae*	9/14	3.4	–168.0 ± 73.6	20.4	57	0	1
*S. sonnei*	8/14	4.2	–154.9 ± 69.2	19.1	54	0	1
*Pseudomonas aeruginosa* sample number 5-8, 13-16, and 27-28							
Species	IF	E[R]	E[ln (*E*_*u*_)]	E[WPC]	E[NIP]	E[NUP]	E[CI]
*P. aeruginosa*	10/10	1.0	–526.3 ± 557.5	59.9	94	23	1
*A. KKS102*	1/10	2.0	–26.0 ± 0.0	2.0	4	2	4
*P. stutzeri*	1/10	2.0	–16.1 ± 0.0	1.8	7	0	2
*E. 638 tid399742*	1/10	3.0	–7.5 ± 0.0	1.2	5	0	2
*E. asburiae*	1/10	5.0	–7.1 ± 0.0	1.2	6	0	2
*Salmonella enterica* sample number 17-20							
Species	IF	E[R]	E[ln (*E*_*u*_)]	E[WPC]	E[NIP]	E[NUP]	E[CI]
*S. enterica*	4/4	1.0	–206.7 ± 15.2	24.7	61	4	1
*S. bongori*	1/4	2.0	–157.3 ± 0.0	19.6	56	0	1
*H. mediterranei*	2/4	3.0	–7.6 ± 0.8	1.0	1	1	11
*C. metallidurans*	4/4	2.5	–6.7 ± 2.9	1.1	2	1	8
*P. FO BEG1*	1/4	4.0	–5.5 ± 0.0	1.0	1	0	10

^a^ The numerical entries in the table are the expected values E[X]. The E[ln (*E*_*u*_)] is followed by its standard deviation ± *σ*_*X*_

## Discussion

Evidently, the clustering procedure employed requires a suitable cutoff *ρ*_*c*_ for making the decision whether or not a genus/species/strain should be clustered with the head genus/species/strain of a cluster. If *ρ*_*c*_ is too small, large clusters are likely to form, making difficult the identification of multiple microorganisms. That is, too small a *ρ*_*c*_ can introduce false negatives. At the other extreme, a large *ρ*_*c*_ can lead to significant identifications of multiple microorganisms even when the sample is made of only one microorganism. That is, too large a *ρ*_*c*_ can introduce false positives. It appears that using 7/8 for *ρ*_*c*_ is a reasonable choice, producing no false positives. Although, the dataset used did not contain any samples of multiple microorganisms, the method proposed might be able to handle such cases albeit a separate independent study must be conducted for verification.

A positive E[NUP] provides important supporting evidence for the identified microorganisms, but it should not be used as the sole evidence. As more protein sequences and genomic sequences become available in biological databases, the E[NUP] value is expected to decrease for most microorganisms. Our study indicates that *E*_*u*_ is a more robust measure than E[NUP]. For example, in Table 4, for the *Mycobacterium tuberculosis H37Rv* samples, the relative difference between the two top ranked species in terms of E[NUP] is small compared with that of E[ln *E*_*u*_]. This demonstrates an advantage of using *E*-values. The computed *E*_*u*_ seems to carry more discriminating power than different quantities based on the number of identified peptides. For this reason, our method uses all identified peptides with *E*-value less than *E*_*c*_ to compute *E*_*u*_.

One difficulty in correctly identifying a microorganism arises from the fact that different microorganisms may have similar proteomes/genomes. This complication intensifies if one tries to separate microbes of highly similar proteomes/genomes, a likely scenario as more proteomes/genomes of newly discovered microorganisms become available. For example, our peptidome approach has no difficulty in identifying the correct *Escherichia* genus while analyzing the PNNL dataset because the inter-genus peptidome similarity is generally weak (see Supplementary Figure S1). However, the substantial similarities among different (sub)strains of *Escherichia Coli* (see Supplementary Figure S2) hinder us from consistently identifying correct strains. One way to alleviate this problem is to utilize additional information to reduce the number of candidate microorganisms in a database. For example, under the assumption that the correct microorganism is in the database, and given that it is pathogenic, we can then bypass all the non-pathogenic microorganisms during identifications. Another scheme that could potentially improve microbial identifications is to combine the results obtained from microbial identification using a MALDI-based or PCR-ESI-MS-based system with the analysis results from an LCMS experiment.

Table 6 shows that for the in-house dataset there is a notable difference in the number of CIPs when allowing up to two MCS versus up to five MCS. This observation is consistent across all samples. This indicates that tryptic digestion of proteins can be improved. Based on recent studies [74, 75], we believe that increasing the digestion time from 15 to 60 min at 50°C can be our next immediate improvement. In addition to increasing the number of CIPs, better tryptic digestion reduces the occurrences of missed cleavages, allowing the analyses to be done with a smaller allowed MCS. This leads to a reduction in number of candidate peptides during database search, which not only improves peptide identification sensitivity but also speeds up the data analyses. Furthermore, shorter tryptic peptides hold fewer protons. This leads to peptides with lower charge states, which not only allows for more accurate charge determinations of the precursor ions but also produces less convoluted *m/z* fragments that are better captured by the scoring functions implemented in current database search tools. The data displayed in Figure 4 indicates that the MS/MS spectra acquired for the PNNL dataset represent the fragmentation spectra of short peptides containing low charges, making peptide identification an easier task for currently available database search tools [54, 62, 63]. The MS/MS spectra for the in-house dataset, however, represent the fragmentation spectra of longer and higher charge-containing peptides, making peptide identification challenging. Panel b of Figure 4 shows that SN1 has a larger number of precursor ions at low charge states than SN9. However, because SN1 contains fewer precursor ions, with molecular weight <6000 Da than SN9, SN1 ends up having a smaller number of CIPs than SN 9 (see Table 6).

It is worthwhile to further discuss what may cause the number of identified peptides from the in-house dataset to be much smaller than that from the PNNL dataset. In addition to the reasons described, we believe that the sample preparation prior to the tryptic digestion as well as additional chromatography/fractionation also contribute. For each PNNL sample, a modified bead beating method was applied to break the cell walls. This customized cell-wall breaking protocol, described earlier, increases the exposure depth of microbial proteome for digestion and thus enables better proteome coverage. In addition, compared with the in-house procedure, we note another difference from the PNNL workflow: many samples were each prefractionated to 24 fractions by strong cation exchange (SCX) chromatography prior to LCMS analysis. This additional chromatography step facilitates better peptide separation and thus promotes a large number of identifications. Evidently, depending on the goal, the optimal protocol varies. In terms of classifications, it is best to optimize the proteome coverage. However, in terms of clinical applications, one may in addition like to minimize the amount of time required for confident identifications.

## Conclusion

In this study, we have proposed a pipeline for microbial identification/classification by processing MS/MS data acquired in a high resolution mass spectrometer. Using a large number of samples from the PNNL dataset, we have shown that the proposed pipeline is able to confidently identify microorganisms at the genus and species levels when the sample preparation was optimized. The importance of an optimized sample preparation is also reflected from the analyses of our in-house *Escherichia* samples, where the correct *Escherichia* genera are often accompanied by *Shigella* because of weak separation in the numbers of observable evidence peptides. These results illustrate that the proposed pipeline can be a useful tool for microorganism identifications if sample preparation is optimized. It should be noted that this pipeline provides accurate *E*-values at the microbial level (*E*_*u*_s). Having accurate statistical significance is advantageous as it provides the correct confidence assignments to the microorganisms identified.

Our results also indicate that microbial identification at the strain level is a challenging task, as the correct strain may not always attain the best rank. This problem will only become harder as the genomes of new microorganisms are sequenced and made available in public databases. To meet the challenge of increasing number of sequenced genomes will require innovations and technological advances in the areas of chromatography, mass spectrometry, statistical analyses, and algorithm developments. That is, a concerted effort of the community is needed.

While we have focused on microbial identifications using samples each composed of one microorganism, in the next phase, we will evaluate how the proposed pipeline performs when using samples containing multiple microorganisms. Our (automated) pipeline for microbial identifications has been implemented in a software tool called MiCId, a command line C++ program. MiCId can be downloaded freely at http://www.ncbi.nlm.nih.gov/CBBresearch/Yu/downloads.html.

## Electronic supplementary material

ESM 1 (PDF 150 kb)
