# Supplementary material for: Identification of Microorganisms by High Resolution Tandem Mass Spectrometry with Accurate Statistical Significance
Source: J Am Soc Mass Spectrom. 2015 Oct 28;27:194–210. doi: 10.1007/s13361-015-1271-2 (PMC4723618; doi:10.1007/s13361-015-1271-2)
Supplement: Supplementary file 1 — (PDF 150 kb) [file 13361_2015_1271_MOESM1_ESM.pdf]

Noname manuscript No.
(will be inserted by the editor)

Electronic Supplementary Material for "Identification of Microorganisms by High Resolution Tandem Mass Spectrometry with Accurate Statistical Significance"

Gelio Alves[1] · Guanghui Wang[2] · Aleksey Y. Ogurtsov[1] · Steven K. Drake[3] · Marjan Gucek[2] · Anthony F. Suffredini[3] · David B. Sacks[4] · Yi-Kuo Yu[1⋆]

[1]National Center for Biotechnology Information, National Library of Medicine, National Institutes of Health, Bethesda, MD 20894, USA
[2]Proteomics Core, National Heart, Lung, and Blood Institute, National Institutes of Health, Bethesda, MD 20892, USA
[3]Critical Care Medicine Department, Clinical Center, National Institutes of Health, Bethesda, MD 20892, USA
[4]Department of Laboratory Medicine, Clinical Center, National Institutes of Health, Bethesda, MD 20892, USA

Received: date / Accepted: date

Example application of formula (6) of the main text

To illustrate the use of formula (6) of the main text, we used it to compute the unified P-value for a species S_i. Let species S_i have six evidence peptides ($\{\pi_i\}_{i=1}^{6}$) with E-values ≤ 1 and with the following ordered pairs of database P-value (p_i) and weight (w_i): ($p_1 = 10^{-8}, w_1 = 1$), ($p_2 = 10^{-7}, w_2 = 1/2$), ($p_3 = 10^{-6}, w_3 = 1/2$), ($p_4 = 10^{-5}, w_4 = 1/4$), ($p_5 = 0.5, w_5 = 1/4$), ($p_6 = 0.8, w_6 = 1/4$). This means that peptide π_1 belongs to the proteome of only one species (S_i only), peptides π_2 and π_3 belong to the proteomes of two species (S_i and another species), and peptides π_4, π_5, and π_6 belong to the proteomes of four species (S_i and three other species). Using 0.01 as the database P-value cutoff (P_c), one finds that only the first four peptides meet the cutoff criterion. Using equation (3) of the main text, these four evidence

⋆ *Correspondence to:* Yi-Kuo Yu; *email:*yyu@ncbi.nlm.nih.gov

National Center for Biotechnology Information, National Library of Medicine, NIH, Bethesda, MD 20894, USA

peptides' database P-values are then combined into a new variable

$$\tau = (10^{-8})^1 \times (10^{-7})^{1/2} \times (10^{-6})^{1/2} \times (10^{-5})^{1/4} = 1.778 \times 10^{-16}. \qquad (1)$$

The formula used in the manuscript to compute a unified P-value is given below

$$\begin{aligned} P_u(\tilde{\tau} \leq \tau) = & \frac{M!}{m!(M-m)!} P_c^m (1-P_c)^{M-m} P_t(\tilde{\tau} \leq \tau |\, m, m_{\text{raw}}) \\ & + \sum_{j=m+1}^{M} \frac{M!}{j!(M-j)!} P_c^j (1-P_c)^{M-j} \times \\ & \times \left[\theta(P_c^j - \tau)\, P_t(\tilde{\tau} \leq \tau |\, j, j) + \theta(\tau - P_c^j)\right], \qquad (2) \end{aligned}$$

and from the information above we know that $M = 6$, $m_{raw} = 1 + 2 \times 1/2 + 1/4 = 2.25$, $m = 3$, $P_c = 0.01$ and $\tau = 1.778 \times 10^{-16}$. Substituting these values in eq. (2), one can compute the unified P-value for species S_i

$$\begin{aligned} & P_u(\tilde{\tau} \leq \tau = 1.778 \times 10^{-16}) \\ & = \frac{6!}{3!3!} (0.01)^3 (0.99)^3 P_t(\tilde{\tau} \leq 1.778 \times 10^{-16} |\, 3, 2.25) \\ & \quad + \sum_{j=4}^{6} \frac{6!}{j!(6-j)!} (0.01)^j (0.99)^{6-j} \times \\ & \quad \times \left[\theta((0.01)^j - \tau)\, P_t(\tilde{\tau} \leq 1.778 \times 10^{-16} |\, j, j) + \theta(\tau - (0.01)^j)\right] \\ & = \frac{6!}{3!3!} (0.01)^3 (0.99)^3\ 2.037 \times 10^{-9} + \sum_{j=4}^{6} \frac{6!}{j!(6-j)!} (0.01)^j (0.99)^{6-j} \times \\ & \quad \times \left[\theta((0.01)^j - \tau)\, P_t(\tilde{\tau} \leq 1.778 \times 10^{-16} |\, j, j) + \theta(\tau - (0.01)^j)\right], \\ & = 3.954 \times 10^{-14} + 4.934 \times 10^{-12} \\ & = 4.974 \times 10^{-12}. \end{aligned}$$

Supplementary Tables

Table S1 MS/MS data produced in-house.

Samples from batch one				
SN[a]	File Name	SL[b]	Instrument	CGL(minutes)[c]
Escherichia coli sample number 1−4				
1	E_L_1.mzML	Low	Orbitrap Elite	90
2	E_M_1.mzML	Medium	Orbitrap Elite	90
3	E_H_1.mzML	High	Orbitrap Elite	90
4	E_S_1.mzML	Saturated	Orbitrap Elite	90
Pseudomonas aeruginosa sample number 5−8				
5	P_L_1.mzML	Low	Orbitrap Elite	90
6	P_M_1.mzML	Medium	Orbitrap Elite	90
7	P_H_1.mzML	High	Orbitrap Elite	90
8	P_S_1.mzML	Saturated	Orbitrap Elite	90
Samples from batch two				
SN	File Name	SL	Instrument	CGL(minutes)
Escherichia coli sample number 9−12				
9	E_L_2.mzML	Low	Orbitrap Elite	90
10	E_M_2.mzML	Medium	Orbitrap Elite	90
11	E_H_2.mzML	High	Orbitrap Elite	90
12	E_S_2.mzML	Saturated	Orbitrap Elite	90
Pseudomonas aeruginosa sample number 13−16				
13	P_L_2.mzML	Low	Orbitrap Elite	90
14	P_M_2.mzML	Medium	Orbitrap Elite	90
15	P_H_2.mzML	High	Orbitrap Elite	90
16	P_S_2.mzML	Saturated	Orbitrap Elite	90
Salmonella enterica sample number 17−20				
17	S_L_2.mzML	Low	Orbitrap Elite	90
18	S_M_2.mzML	Medium	Orbitrap Elite	90
19	S_H_2.mzML	High	Orbitrap Elite	90
20	S_S_2.mzML	Saturated	Orbitrap Elite	90
Samples from batch three				
SN	File Name	SL	Instrument	CGL(minutes)
Escherichia coli sample number 21−26				
21	E_1_1_A.mzML	Medium	Orbitrap Elite	90
22	E_1_1_B.mzML	Medium	Orbitrap Elite	90
23	E_1_1_C.mzML	Medium	Orbitrap Elite	90
24	E_1_2_A.mzML	Medium	Orbitrap Elite	90
25	E_1_2_B.mzML	Medium	Orbitrap Elite	90
26	E_1_2_C.mzML	Medium	Orbitrap Elite	90
Pseudomonas aeruginosa sample number 27−28				
27	P_1_1_A.mzML	Low	Orbitrap Elite	90
28	P_1_1_B.mzML	Low	Orbitrap Elite	90

[a] Sample number (SN).
[b] Sample label (SL).
[c] Chromatography gradient length (CGL).

Table S2 MS/MS downloaded from the Pacific Northwest National Laboratory FTP site.

SN[a]	File Name	Instrument	CGL(minutes)[b]
Escherichia coli K-12 sample number 29−39			
29	Ecoli432_R1-rr_18Dec09_Falcon_09-09-14.mzXML	LTQ Orbitrap 2	100
30	Ecoli432_R2_7Dec09_Falcon_09-09-15.mzXML	LTQ Orbitrap 2	99
31	Ecoli432_R3_7Dec09_Falcon_09-09-16.mzXML	LTQ Orbitrap 2	100
32	Ecoli432_R4_15Dec09_Falcon_09-09-16.mzXML	LTQ Orbitrap 2	100
33	Ecoli433_R1_7Dec09_Falcon_09-09-14.mzXML	LTQ Orbitrap 2	99
34	Ecoli433_R2_7Dec09_Falcon_09-09-15.mzXML	LTQ Orbitrap 2	99
35	Ecoli433_R4_13Dec09_Falcon_09-09-16.mzXML	LTQ Orbitrap 2	100
36	Ecoli434_R1_7Dec09_Falcon_09-09-14.mzXML	LTQ Orbitrap 2	99
37	Ecoli434_R2_7Dec09_Falcon_09-09-15.mzXML	LTQ Orbitrap 2	100
38	Ecoli434_R3_7Dec09_Falcon_09-09-16.mzXML	LTQ Orbitrap 2	99
39	Ecoli434_R4_13Dec09_Falcon_09-09-16.mzXML	LTQ Orbitrap 2	100
Mycobacterium tuberculosis H37Rv sample number 40−48			
40	MtbH37Rv_03_run1_28Mar10_Draco_10-01-17.mzXML	LTQ Orbitrap 1	99
41	MtbH37Rv_03_run2_28Mar10_Draco_10-01-15.mzXML	LTQ Orbitrap 1	99
42	MtbH37Rv_03_run3_28Mar10_Draco_10-01-15.mzXML	LTQ Orbitrap 1	99
43	MtbH37Rv_04_run1_26Mar10_Draco_10-01-17.mzXML	LTQ Orbitrap 1	100
44	MtbH37Rv_04_run2_28Mar10_Draco_10-01-15.mzXML	LTQ Orbitrap 1	100
45	MtbH37Rv_04_run3_26Mar10_Draco_10-01-17.mzXML	LTQ Orbitrap 1	99
46	MtbH37Rv_05_run1_26Mar10_Draco_10-01-17.mzXML	LTQ Orbitrap 1	99
47	MtbH37Rv_05_run2_28Mar10_Draco_10-01-17.mzXML	LTQ Orbitrap 1	100
48	MtbH37Rv_05_run3_28Mar10_Draco_10-01-15.mzXML	LTQ Orbitrap 1	99
Salmonella typhimurium ATCC 14028 sample number 49−56			
49	SBEP_STM_286_2C_5Apr10_Falcon_10-01-23.mzXML	LTQ Orbitrap 2	100
50	SBEP_STM_286_2D_15Apr10_Falcon_10-01-24.mzXML	LTQ Orbitrap 2	100
51	SBEP_STM_287_2C_1Apr10_Falcon_10-01-23.mzXML	LTQ Orbitrap 2	100
52	SBEP_STM_287_2D_20Apr10_Falcon_10-01-24.mzXML	LTQ Orbitrap 2	99
53	SBEP_STM_288_2C_5Apr10_Falcon_10-01-23.mzXML	LTQ Orbitrap 2	100
54	SBEP_STM_288_2D_20Apr10_Falcon_10-01-24.mzXML	LTQ Orbitrap 2	99
55	SBEP_STM_289_2C_1Apr10_Falcon_10-01-23.mzXML	LTQ Orbitrap 2	100
56	SBEP_STM_289_2D_15Apr10_Falcon_10-01-24.mzXML	LTQ Orbitrap 2	99
Yersinia pestis CO92 sample number 57−65			
57	SBEP_YPCO_022_R1_16Sep10_Falcon_10-07-37.mzXML	LTQ Orbitrap 2	100
58	SBEP_YPCO_022_R2_23Sep10_Falcon_10-07-37.mzXML	LTQ Orbitrap 2	99
59	SBEP_YPCO_022_R3_21Sep10_Falcon_10-07-40.mzXML	LTQ Orbitrap 2	100
60	SBEP_YPCO_023_R1_16Sep10_Falcon_10-07-37.mzXML	LTQ Orbitrap 2	100
61	SBEP_YPCO_023_R2_21Sep10_Falcon_10-07-37.mzXML	LTQ Orbitrap 2	100
62	SBEP_YPCO_023_R3_16Sep10_Falcon_10-07-40.mzXML	LTQ Orbitrap 2	99
63	SBEP_YPCO_024_R1_16Sep10_Falcon_10-07-37.mzXML	LTQ Orbitrap 2	100
64	SBEP_YPCO_024_R2_23Sep10_Falcon_10-07-37.mzXML	LTQ Orbitrap 2	99
65	SBEP_YPCO_024_R3_16Sep10_Falcon_10-07-40.mzXML	LTQ Orbitrap 2	99
Yersinia pseudotuberculosis PB1 Plus sample number 66−74			
66	SBEP_YSTB_015_R1_28Sep10_Falcon_10-07-37.mzXML	LTQ Orbitrap 2	100
67	SBEP_YSTB_015_R2_28Sep10_Falcon_10-07-39.mzXML	LTQ Orbitrap 2	99
68	SBEP_YSTB_015_R3_10Oct10_Falcon_10-07-40.mzXML	LTQ Orbitrap 2	99
69	SBEP_YSTB_016_R1_24Sep10_Falcon_10-07-37.mzXML	LTQ Orbitrap 2	99
70	SBEP_YSTB_016_R2_24Sep10_Falcon_10-07-39.mzXML	LTQ Orbitrap 2	99
71	SBEP_YSTB_016_R3_29Sep10_Falcon_10-07-40.mzXML	LTQ Orbitrap 2	100
72	SBEP_YSTB_017_R1_28Sep10_Falcon_10-07-37.mzXML	LTQ Orbitrap 2	100
73	SBEP_YSTB_017_R2_24Sep10_Falcon_10-07-39.mzXML	LTQ Orbitrap 2	100
74	SBEP_YSTB_017_R3_29Sep10_Falcon_10-07-40.mzXML	LTQ Orbitrap 2	100
Shewanella oneidensis MR-1 sample number 75−81			
75	QC_Shew_12_01_pt5_d_29Jun12_Jaguar_12-02-27.mzXML	V Orbitrap ETD 04	99
76	QC_Shew_12_01_pt5_c_29Jun12_Jaguar_12-02-26.mzXML	LTQ Orbitrap 3	59
77	QC_Shew_12_01_Run-07_21Jun12_Roc_12-04-08.mzXML	V Orbitrap ETD 04	300
78	QC_Shew_12_01_Run-07_18Jun12_Roc_12-04-08.mzXML	V Orbitrap ETD 04	300
79	QC_Shew_12_01_2p5_b_16Jun12_Polaroid_11-07-67.mzXML	LTQ Orbitrap 3	59
80	QC_Shew_12_01_2p5_b_15Jun12_Polaroid_11-07-67.mzXML	V Orbitrap 05	99
81	QC_Shew_11-06_2p5_a_28May12_Polaroid_11-12-27.mzXML	V Orbitrap 05	99

[a] Sample number (SN).
[b] Chromatography gradient length (CGL).

Table S3 Bacterial identification at the genus level for the in-house dataset batch one.

Escherichia coli sample number 1−4							
Genus	IF	E[R]	E[ln(E_u)]	E[WPC]	E[NIP]	E[NUP]	E[CI]
Escherichia	4/4	1.5	-122.3 ± 70.2	14.9	39	1	1
Shigella	4/4	1.5	-117.4 ± 62.3	14.1	39	0	1
Enterobacter	2/4	5.0	-36.8 ± 24.4	5.1	18	0	1
Enterobacteriaceae	1/4	3.0	-13.9 ± 0.0	1.8	8	0	1
Raoultella	1/4	5.0	-13.2 ± 0.0	1.5	6	0	1
Pseudomonas aeruginosa sample number 5−8							
Genus	IF	E[R]	E[ln(E_u)]	E[WPC]	E[NIP]	E[NUP]	E[CI]
Pseudomonas	4/4	1.0	-323.6 ± 113.1	41.4	60	21	1
Azospira	2/4	2.0	-13.9 ± 4.6	1.6	3	1	5
Thiobacillus	1/4	2.0	-5.6 ± 0.0	1.0	2	1	5
Rothia	1/4	3.0	-4.8 ± 0.0	1.0	1	0	7
Escherichia	1/4	4.0	1.0 ± 0.0	0.5	2	0	2

The numerical entries in the table are the expected values E[X]. The E[ln(E_u)] is followed by its standard deviation $\pm\sigma_X$.

Table S4 Bacterial identification at the genus level for the in-house dataset batch two.

Genus	IF	E[R]	E[ln(E_u)]	E[WPC]	E[NIP]	E[NUP]	E[CI]
Escherichia coli sample number 9−12							
Escherichia	4/4	1.2	-443.2 ± 241.0	51.8	134	2	1
Shigella	4/4	1.8	-418.0 ± 225.2	49.1	133	0	1
Haloferax	1/4	4.0	-6.7 ± 0.0	1.0	1	0	8
Cupriavidus	4/4	3.0	-6.7 ± 2.1	1.1	2	1	8
Rickettsia	1/4	4.0	-4.0 ± 0.0	1.0	1	0	10
Pseudomonas aeruginosa sample number 13−16							
Genus	IF	E[R]	E[ln(E_u)]	E[WPC]	E[NIP]	E[NUP]	E[CI]
Pseudomonas	4/4	1.0	-243.2 ± 23.2	36.6	49	13	1
Rhodopirellula	1/4	2.0	-1.2 ± 0.0	1.0	1	0	4
Methylobacillus	2/4	3.5	-0.8 ± 6.2	0.8	1	0	2
Alicycliphilus	1/4	2.0	-0.6 ± 0.0	0.2	1	0	2
Escherichia	2/4	5.0	-0.5 ± 5.9	0.8	1	0	2
Salmonella enterica sample number 17−20							
Genus	IF	E[R]	E[ln(E_u)]	E[WPC]	E[NIP]	E[NUP]	E[CI]
Salmonella	4/4	1.0	-232.1 ± 21.1	27.1	61	7	1
Haloferax	2/4	2.5	-7.2 ± 0.5	1.0	1	1	10
Pseudovibrio	1/4	2.0	-5.5 ± 0.0	1.0	1	0	11
Cupriavidus	4/4	2.8	-5.3 ± 2.5	1.1	2	1	6
Aliivibrio	3/4	4.0	-1.7 ± 2.3	0.8	2	0	4

The numerical entries in the table are the expected values E[X]. The E[ln(E_u)] is followed by its standard deviation $\pm\sigma_X$.

Table S5 Bacterial identification at the genus level for the in-house dataset batch three.

Escherichia coli sample number 21−26							
Genus	IF	E[R]	E[ln(E_u)]	E[WPC]	E[NIP]	E[NUP]	E[CI]
Escherichia	6/6	1.3	-475.1 ± 315.2	53.7	152	1	1
Shigella	6/6	1.7	-458.8 ± 291.6	51.4	150	0	1
Cupriavidus	6/6	3.3	-7.6 ± 1.9	1.0	2	1	7
Pusillimonas	5/6	3.8	-6.3 ± 3.7	1.0	2	1	7
Mycoplasma	1/6	5.0	-2.6 ± 0.0	1.0	2	0	6
Pseudomonas aeruginosa sample number 27−28							
Genus	IF	E[R]	E[ln(E_u)]	E[WPC]	E[NIP]	E[NUP]	E[CI]
Pseudomonas	2/2	1.0	-1859.9 ± 277.2	200.7	293	101	1
Acidovorax	1/2	2.0	-23.0 ± 0.0	2.0	4	2	3
Methylobacillus	1/2	2.0	-6.0 ± 0.0	1.2	4	0	2
Escherichia	1/2	3.0	-4.1 ± 0.0	1.1	6	0	2
Trichodesmium	1/2	4.0	-3.6 ± 0.0	0.5	2	0	7

The numerical entries in the table are the expected values E[X]. The E[ln(E_u)] is followed by its standard deviation $\pm\sigma_X$.

Table S6 Bacterial identification at the species level for the in-house dataset batch one.

Escherichia coli sample number 1−4							
Species	IF	E[R]	E[ln(E_u)]	E[WPC]	E[NIP]	E[NUP]	E[CI]
E. coli	4/4	2.0	-123.0 ± 70.2	14.9	39	1	1
S. boydii	4/4	2.2	-114.9 ± 62.1	13.7	38	0	1
S. flexneri	4/4	4.0	-108.9 ± 57.4	13.0	36	0	1
S. sonnei	4/4	3.8	-107.5 ± 55.4	13.0	36	0	1
S. dysenteriae	4/4	4.5	-104.5 ± 54.8	12.7	35	0	1
Pseudomonas aeruginosa sample number 5−8							
Species	IF	E[R]	E[ln(E_u)]	E[WPC]	E[NIP]	E[NUP]	E[CI]
P. aeruginosa	4/4	1.0	-293.4 ± 90.1	35.1	54	15	1
P. stutzeri	1/4	2.0	-16.1 ± 0.0	1.8	7	0	2
A. oryzae	3/4	3.0	-6.7 ± 10.3	1.2	2	1	3
R. dentocariosa	1/4	4.0	-5.5 ± 0.0	1.0	1	0	9
T. denitrificans	1/4	2.0	-4.8 ± 0.0	1.0	2	1	4

TThe numerical entries in the table are the expected values E[X]. The E[ln(E_u)] is followed by its standard deviation $\pm\sigma_X$.

Table S7 Bacterial identification at the species level for the in-house dataset batch two.

Escherichia coli sample number 9−12							
Species	IF	E[R]	E[ln(E_u)]	E[WPC]	E[NIP]	E[NUP]	E[CI]
E. coli	4/4	1.2	-438.9 ± 235.4	51.4	134	2	1
S. boydii	2/4	1.5	-190.3 ± 4.4	24.1	65	0	1
S. dysenteriae	2/4	3.0	-182.4 ± 1.2	23.1	61	0	1
S. flexneri	2/4	4.5	-178.0 ± 4.3	23.4	63	0	1
S. sonnei	2/4	4.5	-173.6 ± 4.6	22.4	61	0	1
Pseudomonas aeruginosa sample number 13−16							
Species	IF	E[R]	E[ln(E_u)]	E[WPC]	E[NIP]	E[NUP]	E[CI]
P. aeruginosa	4/4	1.0	-217.8 ± 20.9	30.9	46	9	1
A. denitrificans	1/4	2.0	-0.6 ± 0.0	0.2	1	0	2
R. baltica	1/4	2.0	-0.5 ± 0.0	1.0	1	0	5
A. aromaticum	1/4	2.0	-0.4 ± 0.0	0.5	2	0	2
A. KH32C	1/4	3.0	-0.4 ± 0.0	0.5	2	0	2
Salmonella enterica sample number 17−20							
Species	IF	E[R]	E[ln(E_u)]	E[WPC]	E[NIP]	E[NUP]	E[CI]
S. enterica	4/4	1.0	-206.7 ± 15.2	24.7	61	4	1
S. bongori	1/4	2.0	-157.3 ± 0.0	19.6	56	0	1
H. mediterranei	2/4	3.0	-7.6 ± 0.8	1.0	1	1	11
C. metallidurans	4/4	2.5	-6.7 ± 2.9	1.1	2	1	8
P. FO BEG1	1/4	4.0	-5.5 ± 0.0	1.0	1	0	10

The numerical entries in the table are the expected values E[X]. The E[ln(E_u)] is followed by its standard deviation $\pm\sigma_X$.

Table S8 Bacterial identification at the species level for the in-house dataset batch three.

Escherichia coli sample number 21−26							
Species	IF	E[R]	E[ln(E_u)]	E[WPC]	E[NIP]	E[NUP]	E[CI]
E. coli	6/6	1.2	-475.1 ± 314.0	53.5	152	1	1
S. boydii	4/6	2.2	-260.7 ± 42.6	31.4	92	0	1
S. flexneri	4/6	4.0	-247.2 ± 41.1	30.6	90	0	1
S. dysenteriae	3/6	2.3	-243.0 ± 33.7	29.0	83	0	1
S. sonnei	2/6	5.0	-231.0 ± 48.6	28.1	84	0	1
Pseudomonas aeruginosa sample number 27−28							
Species	IF	E[R]	E[ln(E_u)]	E[WPC]	E[NIP]	E[NUP]	E[CI]
P. aeruginosa	2/2	1.0	-1609.2 ± 255.5	167.3	272	67	1
A. KKS102	1/2	2.0	-26.0 ± 0.0	2.0	4	2	4
E. 638 tid:399742	1/2	3.0	-7.5 ± 0.0	1.2	5	0	2
E. asburiae	1/2	5.0	-7.1 ± 0.0	1.2	6	0	2
E. cloacae	1/2	6.0	-6.9 ± 0.0	1.7	7	0	2

The numerical entries in the table are the expected values E[X]. The E[ln(E_u)] is followed by its standard deviation $\pm\sigma_X$.

Supplementary Figures

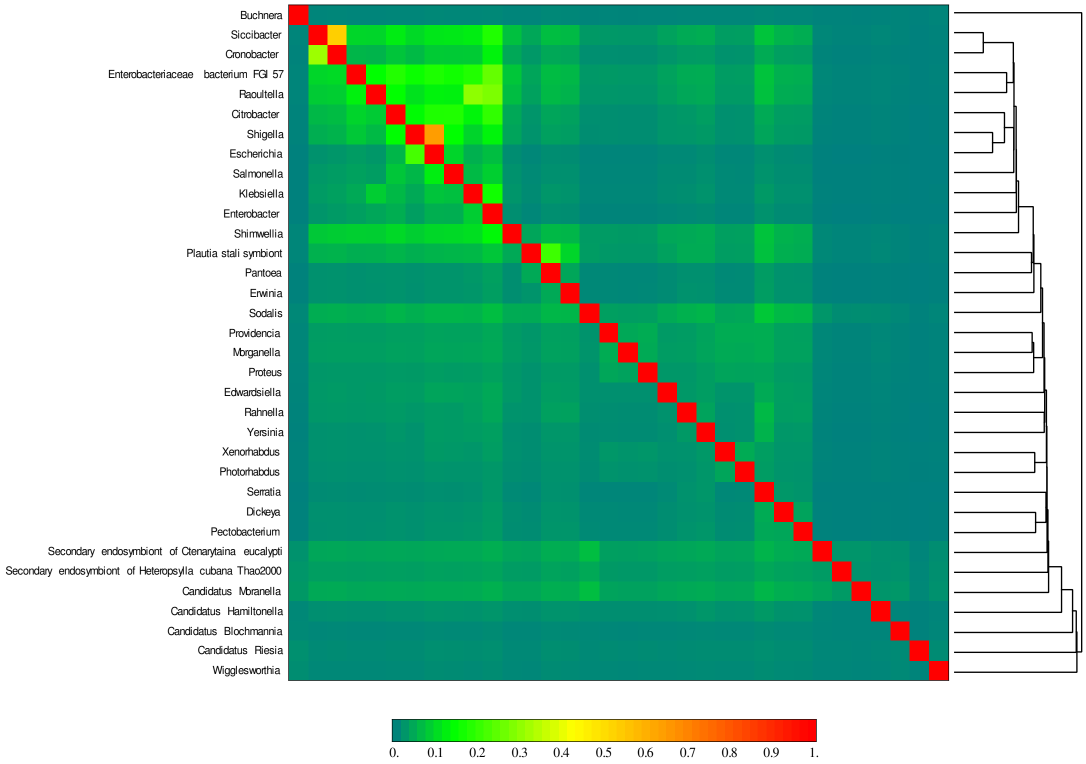

Fig. S1 Inter-genus peptidome similarity and genus identification. Each peptide in the database (see main text) is weighted by the number of C-terminal residues prior to the first potential tryptic cleavage site (K or R). For example, the peptide GPTKLVMQR has weight 5 since there are five amino acids in the LVMQR subsequence. For any two genera i and j, the sum of their shared tryptic peptides' weights is called $S_{i,j}$. Of course, $S_{i,j} = S_{j,i}$. For every genus i, we also sum its tryptic peptides' weights and call it W_i. The (i, j) grid shown in the figure displays in color the value $S_{i,j}/W_i$ (using the scale shown), indicating how similar genus i is to genus j in terms of tryptic peptidome. The reason for weighting the peptides this way is to avoid unwanted redundancy. This heat map shows that there is little inter-genus similarity except for two cases: *Shigella* appears to be similar to *Escherichia* and *Siccibacter* appears to be somewhat similar to *Cronobacter*. This partly explains why it is effective to use peptidomes for correct genus identification.

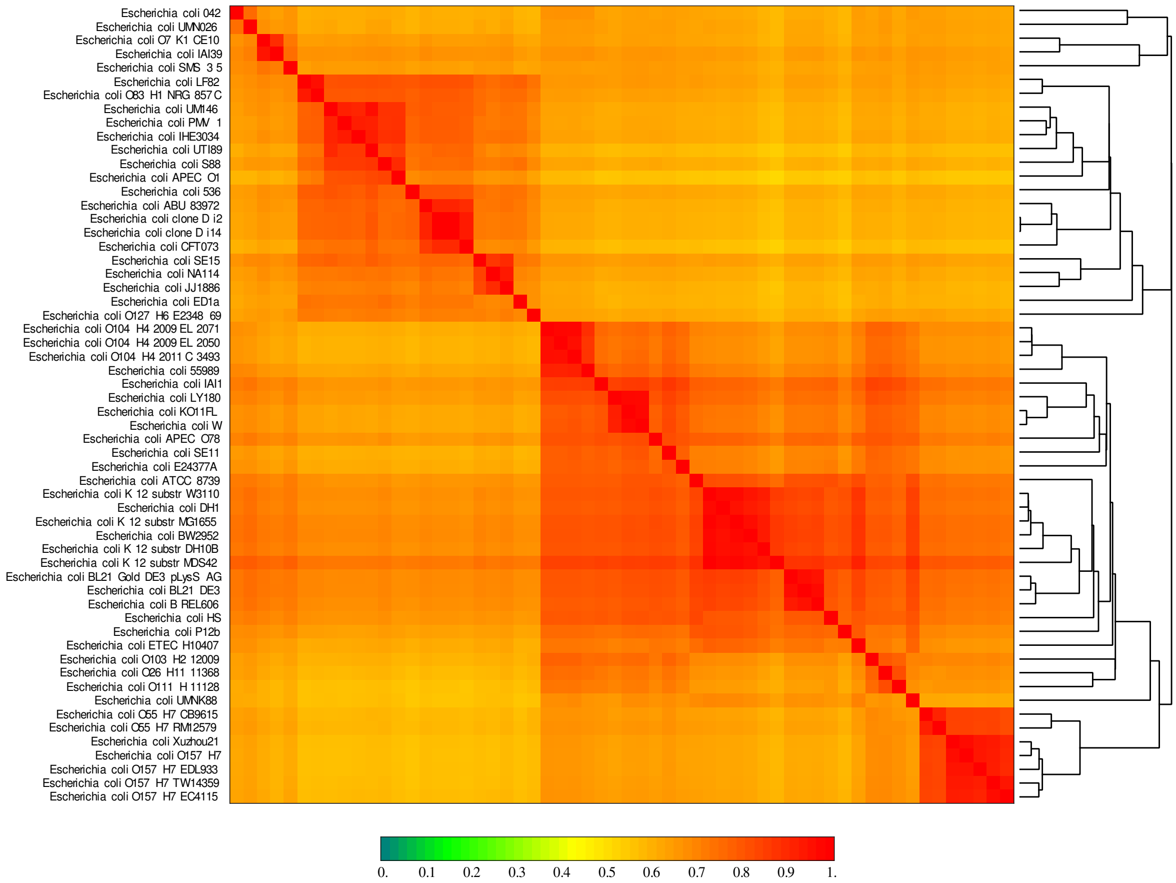

Fig. S2 Inter-strain peptidome similarity and strain identification. Each peptide in the database (see main text) is weighted according to the caption of Figure S1. To investigate why it is difficult to use the peptidome approach to identify correct strains, we include the 58 (sub)strains of *Escherichia Coli* in this heat map. For any two (sub)strains i and j, the sum of their shared tryptic peptides' weights is called $S_{i,j}$. Of course, $S_{i,j} = S_{j,i}$. For every (sub)strain i, we also sum its tryptic peptides' weights and call it W_i. The (i, j) grid shown in the figure displays in color the value $S_{i,j}/W_i$ (using the scale shown), indicating how similar (sub)strain i is to (sub)strain j in terms of tryptic peptidome. This heat map shows that there is little discrimination among strains and almost no distinction among substrains of the same strain. This partly explains the difficulty of consistently identifying correct strains when using the peptidome approach alone.
